# Differential Biological Effects of Dietary Lipids and Irradiation on the Aorta, Aortic Valve, and the Mitral Valve

**DOI:** 10.3389/fcvm.2022.839720

**Published:** 2022-02-28

**Authors:** Nathalie Donis, Zheshen Jiang, Céline D'Emal, Alexia Hulin, Margaux Debuisson, Raluca Dulgheru, Mai-Linh Nguyen, Adriana Postolache, François Lallemand, Philippe Coucke, Philippe Martinive, Marielle Herzog, Dorian Pamart, Jason Terrell, Joel Pincemail, Pierre Drion, Philippe Delvenne, Alain Nchimi, Patrizio Lancellotti, Cécile Oury

**Affiliations:** ^1^Laboratory of Cardiology, Department of Cardiology, GIGA Institute, University of Liège Hospital, CHU Sart Tilman, Liège, Belgium; ^2^Department of Radiotherapy, CHU of Liège, Liège, Belgium; ^3^Department Radiation Oncology, Institut Jules Bordet, Université Libre Bruxelles, Brussels, Belgium; ^4^Belgian Volition Société à Responsabilité Limitée, Gembloux, Belgium; ^5^Department of Oncology and Livestrong Cancer Institutes, Dell Medical School, University of Texas at Austin, Austin, TX, United States; ^6^Volition America, Austin, TX, United States; ^7^Clinical Chemistry, CHU of Liège, Liège, Belgium; ^8^Experimental Surgery Unit, Centre de Recherche du Département de Chrirurgie, Groupe Interdisciplinaire de Géno-Protéomique Appliquée Institute, University of Liège, Liège, Belgium; ^9^Department of Pathology, Centre Hospitalier Universitaire of Liège, Liège, Belgium; ^10^Laboratory of Experimental Pathology, GIGA Institute, University of Liège, Liège, Belgium; ^11^Gruppo Villa Maria Care and Research, Maria Cecilia Hospital, Cotignola, Italy; ^12^Anthea Hospital, Bari, Italy

**Keywords:** cholesterol, palmitic acid, cardiovascular diseases, arteries, heart valves, dietary lipids

## Abstract

**Aims:**

Dietary cholesterol and palmitic acid are risk factors for cardiovascular diseases (CVDs) affecting the arteries and the heart valves. The ionizing radiation that is frequently used as an anticancer treatment promotes CVD. The specific pathophysiology of these distinct disease manifestations is poorly understood. We, therefore, studied the biological effects of these dietary lipids and their cardiac irradiation on the arteries and the heart valves in the rabbit models of CVD.

**Methods and Results:**

Cholesterol-enriched diet led to the thickening of the aortic wall and the aortic valve leaflets, immune cell infiltration in the aorta, mitral and aortic valves, as well as aortic valve calcification. Numerous cells expressing α-smooth muscle actin were detected in both the mitral and aortic valves. Lard-enriched diet induced massive aorta and aortic valve calcification, with no detectable immune cell infiltration. The addition of cardiac irradiation to the cholesterol diet yielded more calcification and more immune cell infiltrates in the atheroma and the aortic valve than cholesterol alone. RNA sequencing (RNAseq) analyses of aorta and heart valves revealed that a cholesterol-enriched diet mainly triggered inflammation-related biological processes in the aorta, aortic and mitral valves, which was further enhanced by cardiac irradiation. Lard-enriched diet rather affected calcification- and muscle-related processes in the aorta and aortic valve, respectively. Neutrophil count and systemic levels of platelet factor 4 and ent-8-iso-15(S)-PGF2α were identified as early biomarkers of cholesterol-induced tissue alterations, while cardiac irradiation resulted in elevated levels of circulating nucleosomes.

**Conclusion:**

Dietary cholesterol, palmitic acid, and cardiac irradiation combined with a cholesterol-rich diet led to the development of distinct vascular and valvular lesions and changes in the circulating biomarkers. Hence, our study highlights unprecedented specificities related to common risk factors that underlie CVD.

## Introduction

Heart diseases are a major health burden worldwide. Coronary artery disease (CAD) is the most prevalent heart disease characterized by the build-up of atherosclerotic plaques in the artery walls, which are responsible for myocardial infarction and stroke, the two leading causes of death (1). Atherosclerosis is a chronic, progressive process that remains asymptomatic for years, making it difficult to diagnose at the early stage. Consequently, atherosclerosis is most often diagnosed following an acute event. Among a population considered at low or intermediate risk of CAD, a study showed that almost 50% of patients had at least one stenotic vascular segment and more than 25% had multiple stenotic segments (2). Percutaneous coronary intervention with stent placement is recommended as the first therapy when available promptly. Afterward, chronic preventive pharmacotherapies are used to reduce the risk of future acute events. The prevalence of atherosclerotic diseases has kept increasing in the last decade, revealing the need to identify new early diagnostic tools, preventive measures, and therapeutic targets.

Valvular heart diseases (VHDs), including calcific aortic valve disease (CAVD) and degenerative mitral valve disease, are other prevalent heart diseases of the aging population (3). These cardiovascular diseases progress very slowly before the onset of the symptom. Hitherto, no pharmacotherapy exists to cure or effectively prevent VHD progression, and valve repair or replacement is the only treatment when a heart valve defect is diagnosed. Although valvular calcification and degeneration are considered active phenomena, the biological mechanisms underlying the VHD remain poorly understood.

While dyslipidemia (lifelong exposure to high cholesterol) is a well-established risk factor for atherosclerosis and CAVD (4–6), statins effectively reduce vascular atherosclerotic diseases (7, 8), but they showed no positive effect on severe CAVD (9, 10). Proprotein convertase subtilisin/kexin type 9 inhibitors are a more recent type of cholesterol-lowering drug with beneficial effects in atherosclerosis (11–13). *In vitro* data showed promising results with these inhibitors on CAVD (14). With regard to mitral valve disease, no studies have ever described a link between dyslipidemia and mitral valve degeneration.

Dietary habits and fatty acid intake are other, less-studied risk factors for heart diseases (15). Fatty acids have different impacts on cardiovascular risk, depending on their saturation degree and C-chain length. For instance, palmitic acid, a main fat component of lard, has been shown to have a deleterious effect on CAD (16). Particularly, our recent study in rabbits indicates that dietary palmitic acid can promote both arterial calcification and CAVD (17).

Beyond dietary fats, radiation therapy to the chest, as a critical component of the treatment regimen of Hodgkin lymphoma, the lung and breast cancer, can also increase the risk of death from cardiac-related causes (18–20). Radiation-induced heart disease (RIHD) is a heterogeneous disease associated with many cardiac manifestations, including CAD and left-sided heart valve diseases (21). The RIHD is a specific entity and the mechanisms underlying its pathogenesis are currently unclear.

This study aimed at evaluating and comparing the impact of the following: (1) two dietary fats, enriched with either cholesterol or lard, as a source of palmitic acid; (2) irradiation in combination with a cholesterol-rich diet, on the aorta and four heart valves. We also examined the interconnexion between circulating biomarkers related to inflammation, oxidative stress, platelet activation, and calcification.

## Materials and Methods

### Ethics Statement and Animal Model

All rabbit experiments conform to the European Union guidelines for the care and use of laboratory animals and were approved by the Animal Ethical Committee of the University of Liège (file number 1951). Seventy-three eight-week-old male New Zealand White rabbits (1.83 ± 0.14 Kg) were purchased from the Charles River, France. The animals were kept in a temperature and humidity-controlled environment in an air-conditioned room with a standard rabbit chow and tap water *ad libitum*. After 2 weeks of acclimation, the rabbits were randomly assigned to seven different groups and diet habituation was conducted for 2 weeks. Protocols started when the rabbits were 12 weeks old and it lasted 16 weeks. Control group (Ctrl, *n* = 13) received a standard chow diet. Vitamin D2 group (Vit.D2, *n* = 7) received a chow diet with 15 days of Vitamin D2 supplementation (ergocalciferol, Sigma-Aldrich) in drinking water at the beginning of the protocol (25,000 U/day/2.5 Kg). The cholesterol-enriched diet group (CHT, n = 17) was treated with 0.3% of the cholesterol-enriched diet and received vitamin D2 (Vit.D2) supplementation as the Vit.D2 group. The lard-enriched diet group (Lard, *n* =13) received 5% of the lard-enriched diet (17) with Vit.D2 supplementation. Irradiation group (IR, *n* = 7) received a chow diet and underwent X-ray cardiac irradiation focused on the aortic valve and the thoracic aorta during the first week of the protocol (24Gy dose divided into 3 fractions of 8Gy delivered at 48-h intervals, chosen to mimic clinically relevant doses used for anticancer radiotherapy) using image-guided radiation on an irradiator specifically made for small animals (X-Rad 225Cx, Precision X-Ray, Inc.). Irradiation combined with cholesterol-enriched diet group (IR/CHT, *n* = 15) received 0.3% of cholesterol-enriched diet plus Vit.D2 supplementation and underwent cardiac irradiation at mid protocol (e.g., after 8 weeks of cholesterol-rich diet). Cholesterol- and lard-enriched diets were produced by Safe (France). Bodyweight was measured on a weekly basis. Blood draws and cardiac CT (X-Rad 225Cx, Precision X-Ray, Inc.) were performed at baseline and at the end of every four weeks (W4, W8, W12, and W16), and aortic wall calcification was visually assessed as absent or present in each of the following segments: ascending aorta, aortic cross, and the descending aorta. An extra blood draw was performed at W2. Anesthesia was induced prior to any procedure (except for body weight measurements) by intramuscular injections of droperidol (0.625 mg/kg), xylazine (5 mg/kg), and ketamine (35 mg/kg) in the hind legs. Euthanasia was conducted in the anesthetized animals by intracardiac injection of pentobarbital (200 mg/kg).

### Histological and Immunofluorescence Analyses of Rabbit Heart

Hearts were harvested, weighed, and fixed in 4% of paraformaldehyde–phosphate-buffered saline (PBS) solution for 24 h before dehydration into 70% of ethanol solution overnight at room temperature (Ctrl: *n* = 6, Vit.D2: *n* = 7, CHT: *n* = 11, Lard: *n* = 6, IR: *n* = 7, IR/CHT: *n* = 7). They were then embedded in paraffin wax and sectioned at 7 μm. Before histological and immunofluorescence analyses, the sections were incubated overnight at 58°C and rehydrated through xylene baths followed by a series of graded ethanol baths. Serial sections were used for the histological assessment of tissue structure on hematoxylin-eosin-stained sections, tissue calcification on alizarin red stained section, and extracellular matrix (ECM) composition on sirius red and Movat's pentachrome-strained sections. The aortic valve area was evaluated by isolating the aortic valve leaflets and measuring their area on multiple serial hematoxylin-eosin-stained sections (4 to 9 per rabbit) by using Adobe Photoshop software. For the immunofluorescence study, the sections were incubated overnight at 58°C prior to chemical rehydration. All sections were treated for 30 min at 96°C with Dako Target Retrieval solution, pH9 (Agilent, S2367) for antigen retrieval, then incubated for 30 min at room temperature with normal goat serum (Abcam, ab7481). Co-staining was conducted by sequential use of monoclonal mouse anti-rabbit RAM-11 primary antibody (Agilent, M0633, 1:50 dilution), TRITC-conjugated goat anti-mouse secondary antibody (Abcam, ab5885, 1:100 dilution), and AlexaFluor 488-conjugated monoclonal mouse anti-alpha smooth muscle actin (αSMA) antibody (ThermoFisher Scientific, #53-9760-82, 1:100 dilution). Nuclei were stained with 4′, 6-diamidino-2-phenylindole (DAPI) (ThermoFisher Scientific, D1306, 1:500,000 dilution). Negative controls corresponding to TRITC-conjugated goat anti-mouse secondary antibody without anti-RAM-11 primary antibody and AF488-conjugated mouse IgG2a kappa isotype control isotype were performed (Supplementary Figure S1). QuPath 0.2.3 software was used to analyze fluorescence staining in the delimitated aortic valve, the mitral valve, and the aorta area. Pixel classifiers were first defined to set a threshold value for each fluorochrome. A script was then applied in batch to automatically count the cell number, mean fluorescence intensities as well as the total tissue area, and the percentages of positive area for each immunostaining.

### RNAseq Analysis

Aorta ring and heart valves were dissected (Ctrl: *n* = 7, CHT: *n* = 6, Lard: *n* = 7, IR/CHT: *n* = 7) at the end of the 16-week protocols, snap frozen in liquid nitrogen, and stored at −80°C until RNA extraction. Total RNA was extracted by the use of miRNeasy Mini Kit (Qiagen) according to the manufacturer's instructions. The integrity of the RNA sample was assessed on a Bioanalyser 2,100 with RNA 6,000 Nano and RNA 6,000 Pico chips (Agilent Technologies). The RNA libraries were prepared with 10 ng of RNA using the SMARTer® Stranded Total RNA-Seq Kit v2 - Pico Input Mammalian (Takara Bio USA, Inc.) according to the manufacturer's instructions. The RNA quantification and normalization were performed with the KAPA Library Quantification kit (comprising P5-P7 primers) on the ABI 7900HT system (ThermoFisher Scientific). Sequencing of the libraries was performed on a NovaSeq6000 System (Illumina) with an S4 reagent kit (300 cycles) and 1.2 nM library loading.

Paired FastQ files for all the samples were assessed for Quality Control using FastQC and then they were processed through nf-core rnaseq pipeline v1.4.2 (https://nf-co.re/rnaseq/1.4.2), with default parameters except for -clip_R2 = 3 and -nextseq-trim = 10, in order to perform mapping, quantification, and QC, and to produce the gene count matrix. The reference genome used was Oryctolagus cuniculus OryCun2.0 with annotation from Ensembl release 99 (http://jan2020.archive.ensembl.org/index.html). Gene counts were normalized by the default method (median of ratios) in the DESeq2 package (22). Gene expressions in each diet or treatment group were compared to the control group by DESeq2; a total of 29,587 tests were performed for each comparison and were followed by Benjamini and Hochberg multiple test correction. An adjusted *p*-value (FDR) less than 0.05 and a fold-change greater than 1.5 were used to highlight a significant difference in gene expression between the two groups. Human orthologs of differentially expressed genes (DEGs) were identified by the online tool, Better Bunny (23) with an identity of 50% as a threshold (24), and were ranked by their π-value calculated with a log-fold change (LFC) and the adjusted *p-*value (25). TopGO package was used to perform gene ontology (GO) analysis with a ranked gene list. The whole RNA-seq analysis was performed in R (version 4.0.4; R Foundation for Statistical Computing, Vienna, Austria).

### Blood Processing

At each blood draw, differential blood cell counts (white blood cells, red blood cell indices, and platelets) were measured in 3.8% of citrated whole blood on a Cell-Dyn 3700 hematocytometer equipped with veterinary software (Abbott Laboratories). Serum aliquots were prepared after 30-min resting to allow for blood clotting followed by centrifugation at 1,700 *g* and room temperature. EDTA and 3.8% citrated plasma aliquots were prepared by two successive centrifugations at 1,700 *g* and room temperature maximum of 1 h after blood draw. Serum and plasma samples were stored at −80°C until further analysis.

### Biomarker Measurements

Measurements of the levels of total cholesterol, triglycerides, LDL-c, HDL-c, calcium, phosphorous, iron, albumin, AST, ALT, creatinine, and urea were performed in serum samples on an AU480 Chemistry Analyzer (Beckman Coulter). Serum levels of IL-6 and plasma [ethylenediamine tetraacetic acid (EDTA)-anticoagulated plasma] levels of DKK1 and PF4 were measured by enzyme-linked immunosorbent assays according to the manufacturer's recommendations (Cusabio). The ent-8-iso-15(S)-PGF2α levels were measured in EDTA-plasma by the ultra-high performance liquid chromatography coupled to tandem mass spectrometry detection. Levels of circulating H3.1-nucleosomes and citrullinated histone H3R8 nucleosomes (H3R8cit-nucleosome), an epigenetic modification, were assessed by blinded Nu.Q® chemiluminescence immunoassays (Volition SRL, Belgium) in EDTA-plasma (Ctrl: *n* = 7–13, Vit.D2: *n* = 7, CHT: *n* = 10–15, Lard: *n* = 7–12, IR: *n* = 7, IR/CHT: *n* = 15).

### Statistical Analysis

Between-treatment comparisons of all variables were performed by ANOVA and *post-hoc* Dunnett test on log transformed data, except for leaflet area quantification, nested GLM model, and *post-hoc* SIDAK tests were performed. Over time changes between two time points in the blood cells and circulating biomarkers were assessed by Wilcoxon signed-rank test. Data are presented as median [interquartile range (p25–p75)]. All tests were performed 2-sided and *P-value* ≤ 0.05 was considered significant. All statistical analyses were performed using SAS 9.4 (SAS Institute, Cary NC).

## Results

### Dietary Cholesterol and Palmitic Acid Induce Distinct Histological Lesions in the Aorta, Aortic Valve, and Mitral Valve

**Figure 1:**
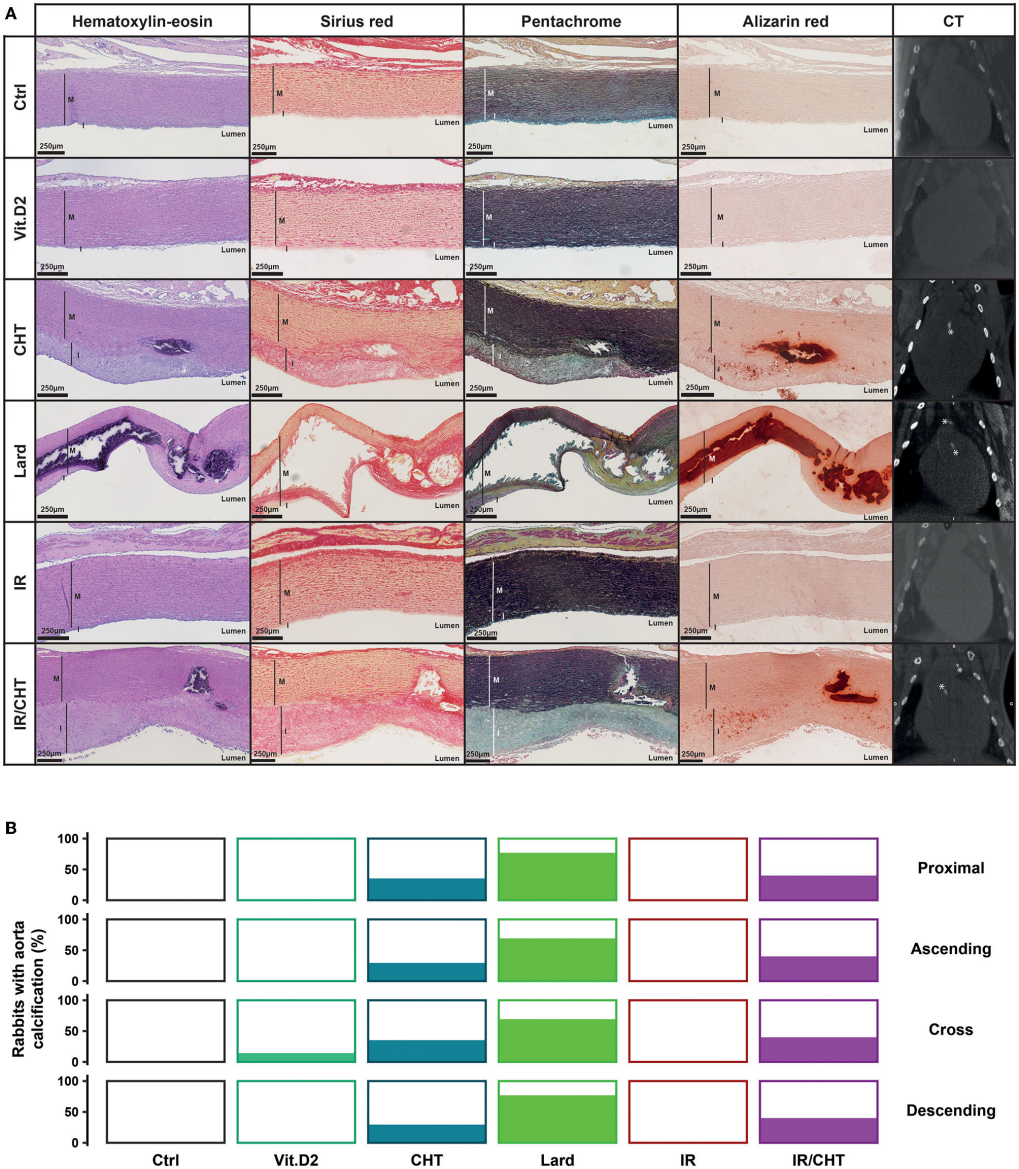
Effect of lard- and cholesterol-enriched diets on the aorta structure. **(A)** Representative pictures of aortic wall sections stained with hematoxylin-eosin, sirius red, Movat's pentachrome, and alizarin red, and CT imaging per rabbit group as indicated. Scale bars = 250 μm. Calcifications observed by CT are indicated by asterisks. **(B)** Percentage of rabbits from each group that presented CT-detected calcification in the different portions of the aorta (Ctrl, *n* = 13; Vit.D2, *n* = 7; CHT, *n* = 17; Lard, *n* = 13; IR, *n* = 7; IR/CHT, *n* = 15). I, indicates intima; M, indicates media.

Rabbits fed for 16 weeks with lard- or cholesterol-enriched diets supplemented with Vit.D2 displayed aortic wall structural alterations that were not observed in rabbits fed with chow diet, or in those receiving the chow diet supplemented with Vit.D2 (Figure 1A). Alizarin red staining of heart sections from rabbits treated with a lard-enriched diet revealed massive media calcification with collagen fiber accumulation and disorganization around the calcific lesions as shown by Sirius Red and Movat's Pentachrome staining. The CT imaging revealed aortic wall calcifications in 70 to 80% of rabbits fed with lard depending on considered aorta segments (Figure 1B). Such macrocalcifications were not observed in Ctrl or Vit.D2 groups, except for one rabbit treated with Vit.D2 which developed calcification in the aortic cross.

In agreement with previous studies (26), rabbits fed for 16 weeks with a cholesterol-enriched diet developed atherosclerotic lesions. Hematoxylin-eosin staining of aortic sections showed intima thickening with cell and lipid infiltration, whereas Sirius Red and Movat's Pentachrome showed disorganized ECM component deposition (collagen fibers and glycosaminoglycans, GAGs). Alizarin red staining revealed calcification nodules at the intima-media junction and diffuse microcalcifications in the thickened intima. Approximately 35% of rabbits fed with a cholesterol-enriched diet displayed calcifications detected by a CT (Figure 1B). We also assessed the infiltration of the aortic wall by macrophages (or macrophage-like cells) by immunodetection of the rabbit RAM-11 marker (27). As described (28), a cholesterol-enriched diet-induced significant infiltration of RAM-11 positive cells in atheroma as compared to Vit.D2 alone (**Figure 4C**).

**Figure 2:**
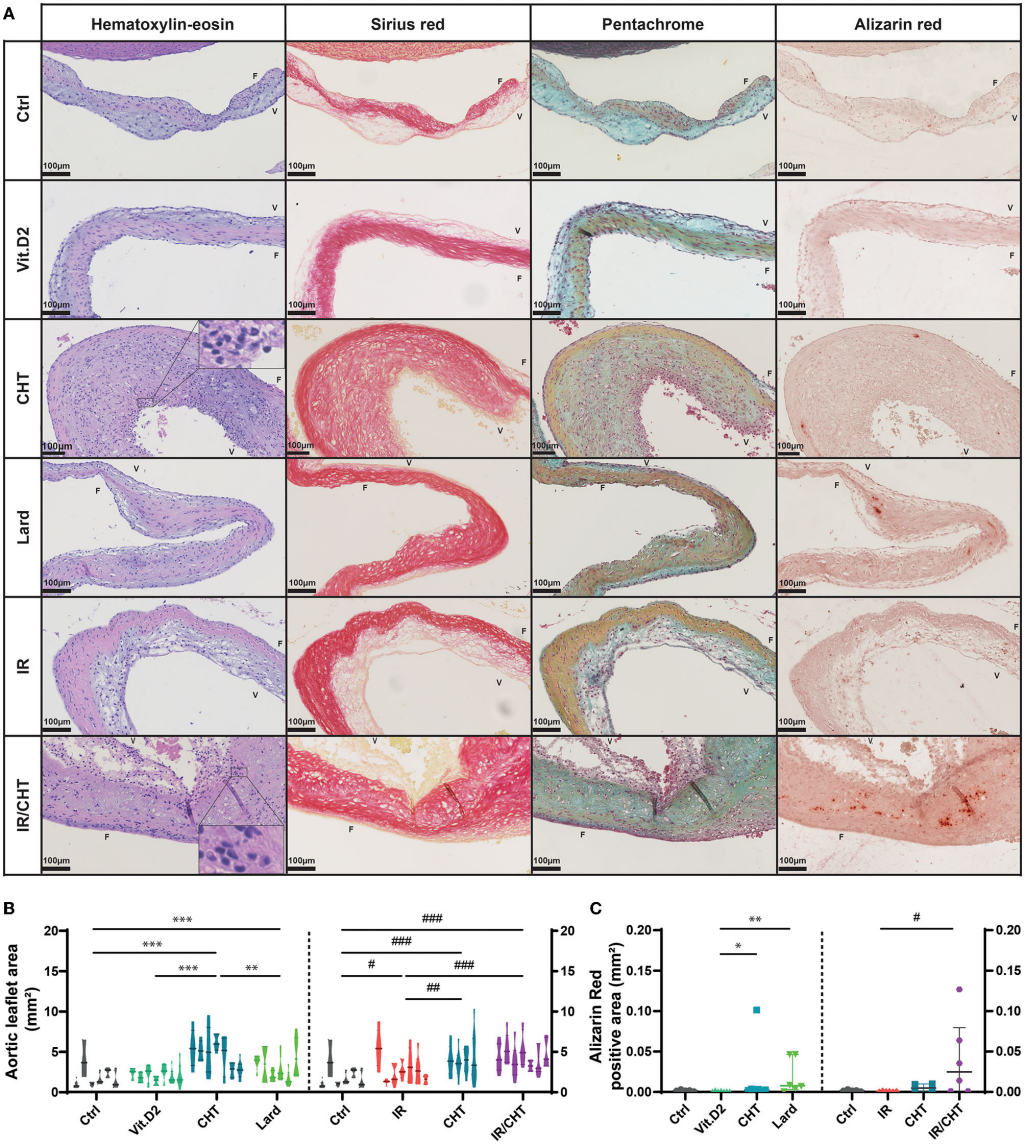
Effect of lard- and cholesterol-enriched diets on the aortic valve leaflet histology. **(A)** Representative pictures of aortic valve leaflet sections stained with hematoxylin-eosin, sirius red, Movat's pentachrome, and alizarin red per rabbit group as indicated. Magnified areas depict neutrophil infiltrates in valvular ventricularis. Scale bars = 100 μm. **(B)** Violin plots represent aortic valve leaflet area measured on hematoxylin-eosin-stained aortic valve sections (each violin box represents one rabbit, 4–8 slides per rabbit). Four-group comparisons were performed by using a nested-GLM model and *post-hoc* SIDAK test on log-transformed data. **(C)** The graph represents the positive area for Alizarin Red staining on the aortic valve sections. Data are presented as medians with P25-P75 interquartiles. Four-group comparisons were performed by ANOVA and *post-hoc* Dunnett's test on log-transformed data (Left: Ctrl, *n* = 5; Vit.D2, *n* = 7; CHT, *n* = 7; Lard, *n* = 6; Right: Ctrl, *n* = 5; IR, *n* = 6; CHT, *n* = 4; IR/CHT, *n* = 6). *P ≤ 0.05; **P ≤ 0.01; ***P ≤ 0.001; ^#^*P* ≤ 0.05; ^##^*P* ≤ 0.01; ^###^*P* ≤ 0.001. F, indicates fibrosa side; V, indicates ventricular side.

**Figure 3:**
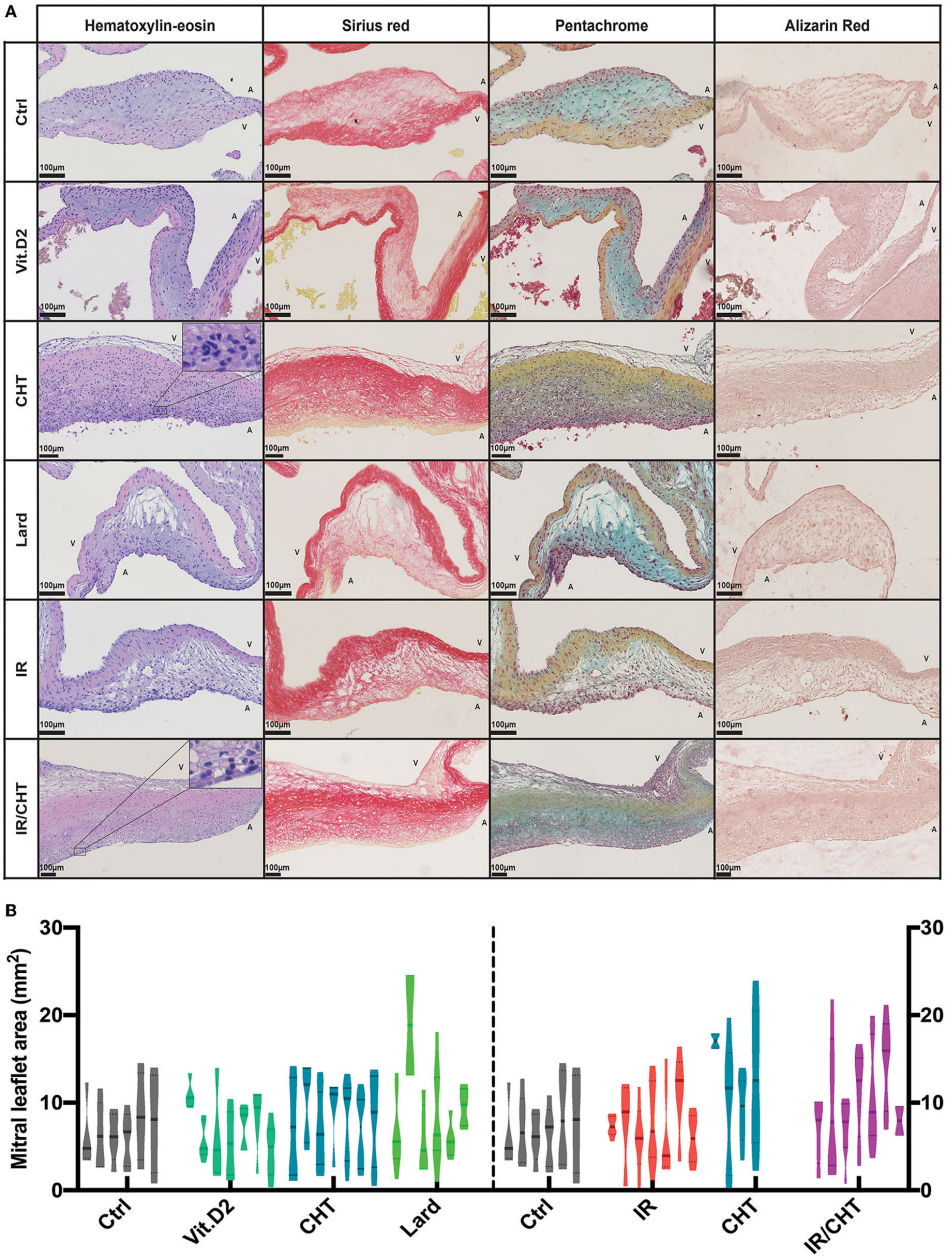
Effect of cholesterol-enriched diets on the mitral valve leaflet histology. **(A)** Representative pictures of mitral valve leaflet sections stained with hematoxylin-eosin, sirius red, and Movat's pentachrome per rabbit group as indicated. Magnified areas depict neutrophil infiltrates in valvular atrialis. Scale bars = 100 μm. **(B)** Violin plots represent mitral valve leaflet area measured on the histological mitral valve sections (each violin box represents one rabbit, 4–8 slides per rabbit) stained with hematoxylin-eosin. Four-group comparisons were performed by using a nested-GLM model and *post-hoc* SIDAK test on log-transformed data. *P* = non-significant. A, indicates atrial side; V, indicates ventricular side.

**Figure 4:**
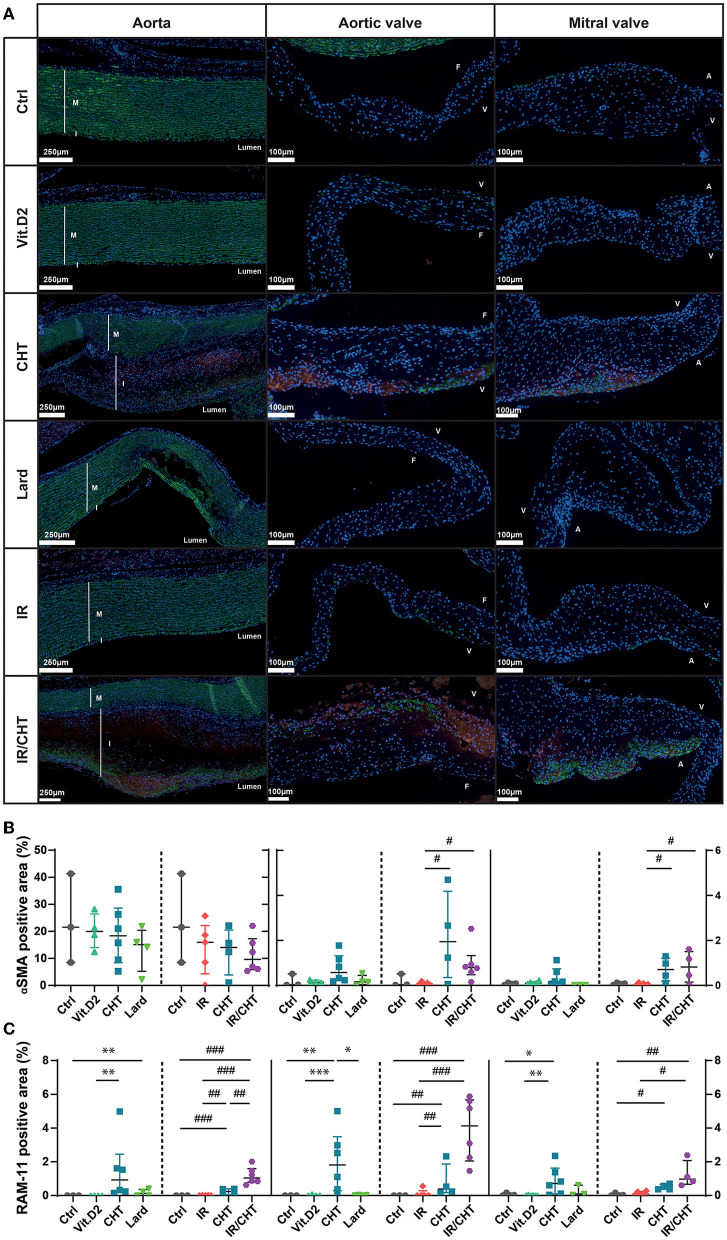
Immuno-detection of RAM-11- and αSMA positive cells in the aorta and heart valves. **(A)** Representative pictures of RAM-11 and αSMA immunofluorescence costaining performed on the aorta, the aortic valve, and the mitral valve sections. Nuclei are stained with DAPI and appear in blue. αSMA- and RAM-11-positive cells are shown in green and red, respectively. Scale bars = 250 μm for aorta and 100 μm for the aortic and mitral valves. **(B)** Quantification of αSMA-positive cells in terms of fluorescence area percentage in the aorta (left), aortic valve (middle), and the mitral valve (right). **(C)** Quantification of RAM-11-positive cells in terms of fluorescence area percentage in the aorta (left), aortic valve (middle), and the mitral valve (right). Data are presented as median with P25–P75 interquartiles. Four-group comparisons were performed by ANOVA and *post-hoc* Dunnett's test on log-transformed data. Aorta: left: Ctrl, *n* = 3; Vit.D2, *n* = 4; CHT, *n* = 6; Lard, *n* = 4; right: Ctrl, *n* = 3; IR, *n* = 5; CHT, *n* = 4; IR/CHT, *n* = 6. Aortic valve: left: Ctrl, *n* = 3; Vit.D2, *n* = 6; CHT, *n* = 6; Lard, *n* = 3; right: Ctrl, *n* = 3; IR, *n* = 5; CHT, *n* = 4; IR/CHT, *n* = 6. Mitral valve: left: Ctrl, *n* = 4; Vit.D2, *n* = 7; CHT, *n* = 7; Lard, *n* = 6; right: Ctrl, *n* = 4; IR, *n* = 6; CHT, *n* = 4; IR/CHT, *n* = 4. *P ≤ 0.05; **P ≤ 0.01; ***P ≤ 0.001; ^#^*P* ≤ 0.05; ^##^*P* ≤ 0.01; ^###^*P* ≤ 0.001. A, atrial side; F, fibrosa side; I, intima; M, media; V, ventricular side.

Alterations of the aortic valve structure were observed in the two diet groups (Figure 2A). In the cholesterol group, the aortic valve leaflets were significantly thickened with sparse calcification spots, cell infiltration and accumulation of collagen fibers, and GAGs (Figures 2A,B). Immunostaining of RAM-11 and of the muscle-specific αSMA marker revealed significant macrophage infiltration and few αSMA positive cells, both located in valvular ventricularis (Figure 3). In contrast, rabbits fed with a lard-enriched diet displayed calcific nodules in the aortic valve with only slight leaflet thickening compared to cholesterol (Figure 2) and no detectable RAM-11 or αSMA positive cells (Figure 4). No aortic valve alteration was observed in the chow diet and Vit.D2 groups.

The mitral valve was also affected in the cholesterol-enriched diet group (Figure 3), with cellular infiltrates and ECM remodeling in spongiosa, without leaflet thickening. The signal for RAM-11 positive cells was significantly increased as compared to the control and Vit.D2 groups (Figure 4C). As for the aortic valve, only few αSMA positive cells were observed in mitral valves. Rabbits from the chow diet, Vit.D2, and lard groups did not show any visible modification of the mitral valve structure.

None of the diets caused histological modifications of the tricuspid and pulmonary valves (data not shown).

### Cardiac Irradiation Promotes Cell Infiltration in the Aorta, Aortic Valve, and Mitral Valve

Our histological analyses did not reveal any aortic histological alterations when cardiac irradiation was applied in rabbits fed with a chow diet kept for 8 weeks post-irradiation (Figure 1A). However, rabbits fed for 16 weeks with a cholesterol-enriched diet and receiving cardiac irradiation at week 9 displayed more macrocalcification in the aorta than rabbits that were not irradiated. Calcification was detected by cardiac CT in all aortic segments in 40% of rabbits (Figure 1B). Also, the atherosclerotic plaque core of rabbits that received cardiac irradiation combined with cholesterol diet significantly increased in the numbers of RAM-11 positive cells as compared to rabbits fed with a chow diet, a cholesterol diet without irradiation, and in rabbits that received irradiation whilst on chow diet (Figures 4A,C).

The addition of irradiation to the cholesterol-enriched diet induced the aortic valve structure alterations that were not observed in the irradiation-only group. Thickened aortic valve leaflets displayed more cellular infiltrates and RAM-11 positive cells than the control groups (Figures 4A,C). Aortic valves were more calcified than those of rabbits from the cholesterol group (Figures 2A,B).

As for mitral valves, the combination of irradiation with the cholesterol diet led to increased infiltration of RAM-11 positive cells as compared to the irradiation alone (Figure 4C).

### Differential RNA Expression Profiles in the Aorta, Aortic Valve, and Mitral Valve in Response to Lard- and Cholesterol-Enriched Diets

To get insight into the biological mechanisms underlying vascular and valvular modifications, we then performed RNAseq experiments on RNA extracted from the dissected proximal aorta and all cardiac valves. The RNAseq identified 29.587 genes annotated in the rabbit genome, among which 15.135 genes could be assigned to human genes. The cholesterol-rich diet modified the expression of 1.102 (898 unique human orthologs) genes in the aorta, 612 (492) genes in the aortic valve, 281 (229) genes in the mitral valve, and only 4 (3) and 6 (6) genes in the tricuspid and pulmonary valve, respectively. The lard-rich diet yielded much less DEGs than the cholesterol-rich diet, comprising 76 (66) genes in the aorta and 40 (36) genes in the aortic valve. No DEGs were found in the three other cardiac valves in response to the lard diet.

We then assessed the impact of these changes in RNA expression on biological processes in each tissue. To detect the differential effects of each diet in every tissue, both tissue-specific and common DEGs were included in these analyses.

**Figure 5:**
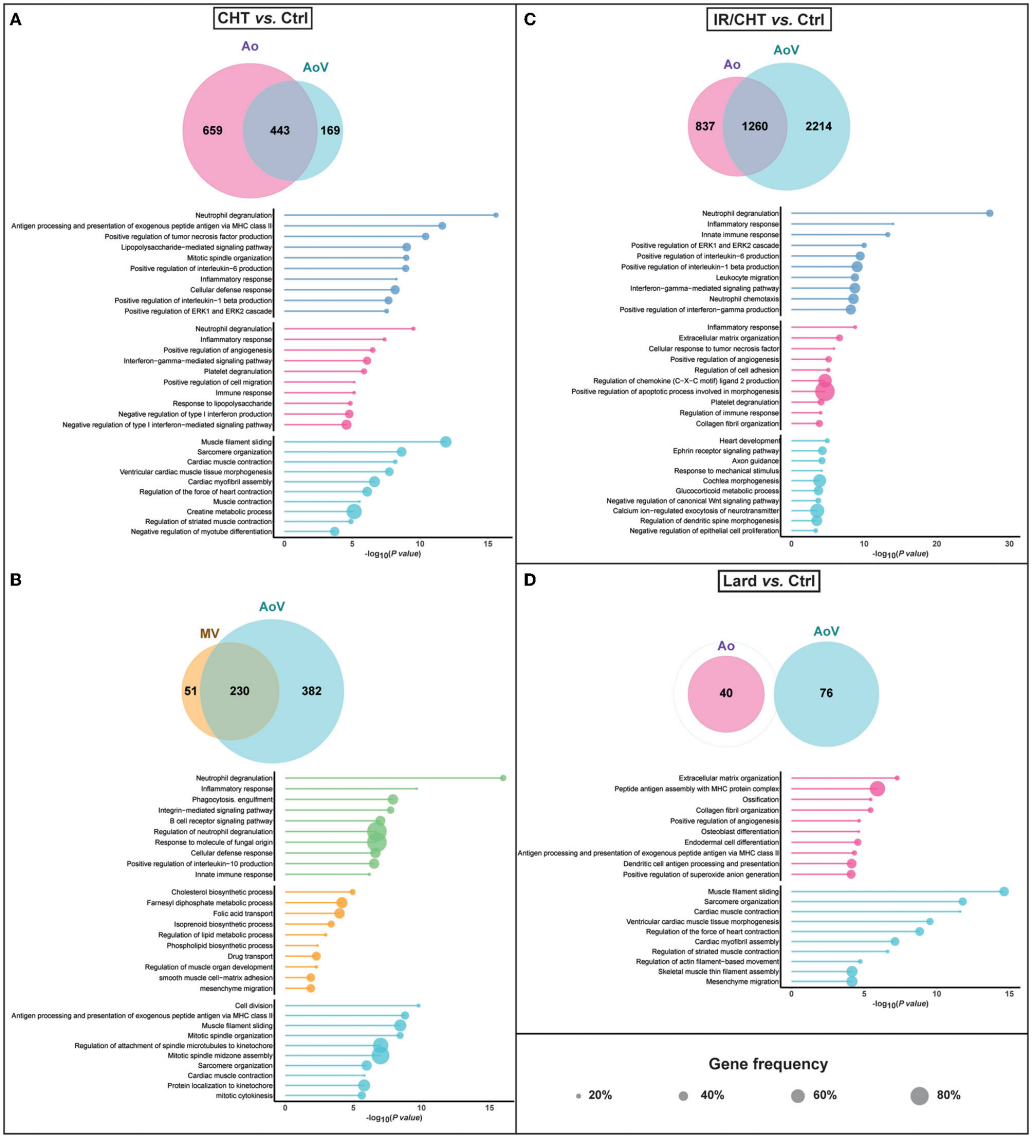
RNA sequencing (RNAseq) analysis of differentially expressed genes (DEGs) in the aorta, the aortic valve, and the mitral valve induced by cholesterol-enriched diet (CHT), alone or combined with irradiation (IR/CHT), and lard-enriched diet as compared to chow diet (Ctrl) (Ctrl, *n* = 7; CHT, *n* = 6; Lard, *n* = 7; IR/CHT, *n* = 7). Venn diagrams represent numbers of specific and common DEGs between tissue pairs for each comparison. Lollipop plots of gene ontology (GO) terms depict the top 10 biological processes affected by specific and common DEGs, with corresponding gene frequency and -log_10_ (*P*-value) (Wald test in DESeq2 package). Detailed statistical methods are provided in the Methods section. **(A)** Comparison of DEGs and affected biological processes in the aorta and aortic valve following 16-week treatment with a cholesterol-rich diet. **(B)** Comparison of DEGs and affected biological processes in the aortic and mitral valves following 16-week treatment with a cholesterol-rich diet. **(C)** Comparison of DEGs and affected biological processes in the aorta and aortic valve following 16-week treatment with a cholesterol-rich diet combined with irradiation. **(D)** Comparison of DEGs and affected biological processes in the aorta and the aortic valve following 16-week treatment with a lard-rich diet. Ao, aorta; AoV, aortic valve; MV, mitral valve.

We first studied the effect of a cholesterol-rich diet on the aorta and aortic valve. Among the 612 DEGs identified in the aortic valve of rabbits from the cholesterol group, 443 DEGs were commonly affected in the aorta (Figure 5A, Supplementary Tables S1–S3). These common DEGs were involved in inflammatory and cellular defense responses, including the production of major inflammatory cytokines and neutrophil degranulation. Mitotic spindle organization was also affected, pointing to an effect on cell division. The 659 aorta-specific DEGs were enriched in genes contributing to additional effects on immune response and signaling pathways, positive regulation of cell migration, angiogenesis, and platelet degranulation, while the 169 aortic valve-specific DEGs were involved in cardiac muscle contraction, tissue morphogenesis, and myofibril assembly. These data indicate that inflammation likely contributes to aortic valve alterations induced by a cholesterol-rich diet similarly as in the aorta, but additional muscle-related processes could also be affected.

We next focused on the comparison between the aortic valve and mitral valve in the cholesterol-fed group. Whereas the DEGs commonly found in the mitral and aortic valves included genes involved in inflammation and cellular defense response, we surprisingly observed that the cholesterol-rich diet mainly affected lipid biosynthetic and metabolic processes as well as folic acid transport in the mitral valve (Figure 5B, Supplementary Tables S4–S6). This finding may thus reveal that not only inflammation but also key metabolic cellular processes underlie the mitral valve alterations in hypercholesterolemia.

We did not identify the genes that were commonly affected in the aorta and aortic valve by the lard-rich diet. The few DEGs that were found to be specific to the aortic valve were all involved in muscle-related processes, including contraction, filament sliding, and sarcomere organization (Figure 5D, Supplementary Tables S7, S8). Intriguingly, these processes were similarly found to be affected by the cholesterol-rich diet, suggesting that dietary cholesterol and palmitate could have a similar biological impact on the aortic valve. In contrast, in the aorta, ECM organization, osteoblast differentiation, and ossification processes were more prevalent in response to lard than cholesterol. Of interest, inflammation would not be the main process induced by the lard diet, which is in sharp contrast to the cholesterol diet.

### Cardiac Irradiation Enhances Changes in the Expression of Inflammation-Related Genes

The addition of irradiation to a cholesterol-rich diet significantly increased the numbers of DEGs both in the aorta and the aortic valve. This was particularly true for the aortic valve. Indeed, 2,097 (1714) and 3,474 (2715) DEGs were identified in the aorta and aortic valve, respectively, which indicated that the aortic valve might be more impacted than the aorta following irradiation. The addition of irradiation to the cholesterol-rich diet modified the expression of 15 (12) additional genes in the aorta and 602 (512) genes in the aortic valve as compared to cholesterol without irradiation. Interestingly, we observed that the common DEGs identified both in the aorta and aortic valve contributed to the same biological processes as a cholesterol-rich diet alone, namely neutrophil degranulation, inflammatory, and immune responses (Figure 5C, Supplementary Tables S9–S11). However, the gene frequency in these processes was doubled by the addition of irradiation, indicating that irradiation could promote inflammation in a neutrophil-dependent manner (Figures 5A,C). Processes, such as neutrophil chemotaxis and leukocyte migration indeed became predominant. In the aortic valve, specific processes were nevertheless affected, such as ephrin receptor and Wnt signaling pathways, heart development, and glucocorticoid metabolism, suggesting that irradiation could disturb aortic valve tissue homeostasis and metabolism. In the aorta, effects on collagen fibril and ECM organization predominated in addition to inflammation and platelet degranulation (Figures 5A,C).

### Circulating Levels of Lipids and Biomarkers Related to Calcification, Inflammation, Oxidative Stress, and Platelet Activation

We wanted to determine whether diet- and irradiation-induced structural and RNA changes could be reflected systemically. For this purpose, we measured the plasma levels of biomarkers related to some of the biological processes identified in our RNAseq dataset. These measurements were performed at baseline and after 16 weeks. We first analyzed differential blood cell counts. Neutrophil count was the only parameter to be increased in the cholesterol-treated rabbits, with or without cardiac irradiation, as compared to Ctrl rabbits (Supplementary Table S12). We then studied the effects of lard-enriched diet and cholesterol-rich diet, combined either with irradiation or without irradiation on the circulating levels of conventional lipid biomarkers (Table 1). As expected, feeding rabbits for 16 weeks with a cholesterol-enriched diet increased their circulating levels of triglycerides, total cholesterol, LDL, and HDL. Surprisingly, rabbits from the irradiation-only group had increased levels of triglycerides. In agreement with our recent study (17), the lard-enriched diet did not induce changes in conventional lipid biomarkers. The hepatic marker, asparate transaminase, increased upon the intake of a cholesterol-enriched diet, whereas the albumin levels were elevated in rabbits receiving cholesterol combined either with or without irradiation (Supplementary Table S13).

**Table 1:** Effects of cholesterol- and lard-enriched diets as well as cardiac irradiation in addition to cholesterol-enriched diet on the circulating levels of conventional lipid biomarkers.

	**CHT**			**Lard**			**IR/CHT**		
	**Pre**	**Post**	***P-value***	**Pre**	**Post**	***P-value***	**Pre**	**Post**	***P-value***
Triglycerides (mg/dl)	78.98 (58.73–99.14)	134.65 (59.31–159.69)	0.010	61.36 (51.39–81.67)	69.67 (53.09–184.29)	0.301	87.15 (67.47–132.15)	172.98 (115.58–296.14)	0.005
Cholesterol (mg/dl)	33.07 (24.57–48.23)	849.50 (846.72–857.80)	<0.0001	42.00 (30.31–47.71)	22.44 (14.93–40.16)	0.052	37.85 (29.77–49.31)	841.43 (836.43–847.31)	<0.0001
LDL (mg/dl)	15.48 (12.24–26.99)	651.34 (550.05–778.28)	<0.0001	19.80 (13.52–26.90)	11.54 (7.25–20.58)	0.204	21.59 (15.47–28.59)	692.38 (600.64–804.07)	<0.0001
HDL (mg/dl)	21.61 (15.158–24.84)	110.36 (74.21–130.27)	<0.0001	25.96 (19.57–28.54)	13.34 (8.54–15.53)	0.016	20.84 (14.63–25.77)	129.28 (91.38–151.54)	<0.0001

*LDL, Low-density lipoprotein; HDL, High-density lipoprotein. P-value: Wilcoxon signed-rank test*.

**Figure 6:**
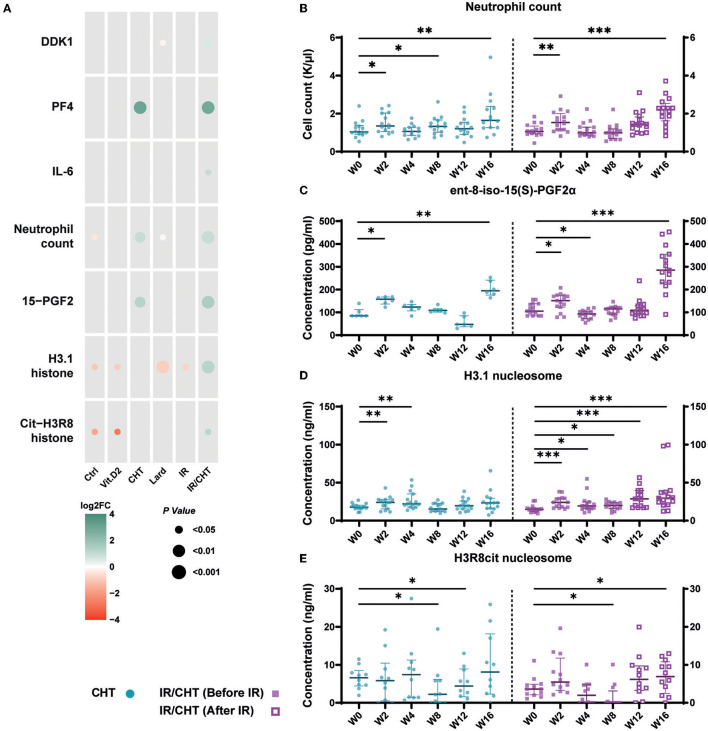
Effect of cholesterol- and lard-enriched diets, and irradiation on the circulating biomarkers of calcification, platelet activation, inflammation, oxidative stress, and on circulating nucleosomes. **(A)** Bubble plot represents concentration fold-change of biomarkers of interest between week 16 (W16) and baseline. Over time evolution of neutrophil count. **(B)** 15-PGF2 **(C)** and circulating nucleosomes **(D,E)**. Graphs represent median with P25-P75 interquartiles. *P ≤ 0.05; **P ≤ 0.01; ***P ≤ 0.001 (Wilcoxon signed-rank test; Ctrl, *n* = 7–13; Vit.D2, *n* = 7; CHT, *n* = 10–15; Lard, *n* = 7–12; IR, *n* = 7; IR/CHT, *n* = 15).

We then analyzed the levels of biomarkers involved in calcification, inflammation, oxidative stress, and platelet activation (Supplementary Figure S2). Rabbits fed for 16 weeks with a lard-rich diet had reduced circulating levels of DKK1, an inhibitor of the Wnt signaling pathway which is involved in the calcification processes (Figure 6A). In contrast, rabbits that received cardiac irradiation combined with a cholesterol diet had elevated levels of DKK1 compared to baseline. Changes in DKK1 levels were neither observed in response to cholesterol diet alone nor in the irradiation alone.

Cholesterol-enriched diet increased the plasma levels of the specific platelet activation marker, platelet factor-4 (PF4), as well as the neutrophil count and the oxidative stress marker, ent-8-iso-15(S)-prostaglandin F2α (ent-8-iso-15(S)-PGF2α) (Figures 6C,E,F). Combined irradiation and a cholesterol-rich diet induced a significant increase in the plasma levels of the major inflammatory cytokine IL-6. as well as the levels of H3.1-nucleosome and citrullinated H3R8-nucleosome (Figure 6A) are consistent with our data of RAM-11 staining and our RNAseq findings on a predominant role of inflammation and neutrophil degranulation in this rabbit group.

Lard-enriched diet did not increase inflammation- or oxidative stress-related biomarkers, in accordance with large, calcified area observed in the aorta and aortic valve without the major implication of inflammatory processes as found in the RNAseq analyses.

To identify possible early biomarkers of lesion development, we performed serial measurements of the neutrophil count, ent-8-iso-15(S)-PGF2α, H3.1-nucleosome, and citrullinated H3R8-nucleosome levels in cholesterol-enriched diet and irradiation combined with cholesterol-enriched diet groups. The neutrophil count increased as early as after 15 days following the dietary cholesterol intake (Figure 6B). Levels of the oxidative stress marker, ent-8-iso-15(S)-PGF2α, were also elevated after 15 days of cholesterol diet (Figure 6C). In the cholesterol diet group, circulating H3.1-nucleosome levels quickly rose after 15 days and 4 weeks and dropped thereafter, while it went on increasing when irradiation was applied at mid-protocol (Figure 6D). Only rabbits that received irradiation combined with a cholesterol diet had increased the circulating levels of citrullinated H3R8-nucleosome after 16 weeks (Figure 6E).

## Discussion

Our study demonstrates differential effects on the vascular and valvular structures induced by cholesterol- and lard-enriched diet, as well as by combining the cholesterol diet with cardiac irradiation.

Cholesterol-rich diet not only induced well described atherosclerotic lesions but also aortic valve thickening. In addition, calcification occurred in the aortic valve leaflets. The two left-sided aortic and mitral valves displayed cellular infiltrates, including macrophages as observed by RAM-11 immunostaining, and excess ECM deposition. In contrast, a lard-rich diet caused massive aortic media calcification with no intima thickening, and the aortic valve calcified without changes in the leaflet thickness and obvious ECM remodeling. The RNAseq analyses of the aorta, aortic valve, and mitral valve revealed distinct predominant biological processes in lesion development depending on the diet. A cholesterol-rich diet mostly triggered inflammation-related processes in all three tissues. Aorta-specific biological processes were also identified that were involved in processes, such as platelet degranulation, angiogenesis, and cell migration, which are well known to contribute to atherogenesis (29–31). The affected aortic valve-specific biological processes were involved in muscle-related processes. Interestingly, inflammation-related biological processes were also enhanced by a cholesterol-enriched diet in the mitral valve, as well as the processes involved in lipid biosynthesis and metabolism. This is consistent with the recent observations of increased infiltration of inflammatory cells and lipids on the histological sections of the mitral valve and their association with serum cholesterol levels (32). In contrast to a cholesterol-rich diet, a lard-enriched diet did not promote inflammatory processes in the aorta, but rather the processes involved in ECM organization, osteoblast differentiation, and ossification. This indicates that a lard-enriched diet, i.e., dietary palmitate intake could impact arteries *via* different mechanisms than a cholesterol-rich diet, leading to distinct structural lesions. Likewise, while muscle-related mechanisms are likely to be involved in the development of aortic valve alterations in response to cholesterol- and lard-enriched diet, inflammatory processes would specifically contribute to the effect of cholesterol diet on the valve.

The addition of cardiac irradiation to a cholesterol-enriched diet led to similar histological lesions in the aorta, the aortic valve, and the mitral valve. However, atherosclerotic plaque and aortic valve and mitral valve leaflets had higher content in RAM-11 positive cells when irradiation was combined with cholesterol-rich diet than with a cholesterol diet alone. Also, the observed increase of αSMA positive cells in the mitral valve suggests an ongoing transdifferentiation process of valvular cells. Valvular interstitial cells could transform into myofibroblasts following cardiac irradiation, which could promote valve remodeling and ECM production (33). These findings support a possible enhancement of tissue inflammation following cardiac irradiation. This proposition was confirmed by our RNAseq data. Indeed, cardiac irradiation doubled the number of genes involved in inflammation-related processes as compared to a cholesterol-enriched diet alone. Neutrophil degranulation was a predominant activated biological process following cardiac irradiation, suggesting a major contribution of this cell type to the RIHD development mechanism. This observation can be of major clinical importance in view of key roles for neutrophils in CVD (34) and a recent report on a link between the severity of aortic stenosis and NETosis (35). Furthermore, the neutrophil-to-lymphocyte ratio has been associated with worse patient survival after chemoradiotherapy (36).

Blood neutrophil count was increased in rabbits fed with cholesterol-enriched diets, combined with cardiac irradiation or without cardiac irradiation. In addition, the circulating levels of IL-6 were higher when cardiac irradiation was added to a cholesterol-rich diet, confirming RNA-seq results of increased inflammation in response to cardiac irradiation. Neutrophil activation could promote vascular and valvular lesions through increased oxidative stress, as suggested by increased plasma levels of ent-8-iso-15(S)-PGF2α, but also through histone-mediated mechanisms as indicated by the increased levels of H3.1 and citrullinated H3R8 nucleosomes. Extracellular histones have been associated with cardiac injury in patients with septic shock (37). They can promote inflammation through activation of the Toll-like receptors, which are implicated in atherosclerosis pathogenesis (38–40). Extracellular histone can mediate lytic cell death and recent findings showed that citrullinated histones can promote foam cell formation *in vitro* (41, 42). The levels of H3.1-nucleosome were increased as early as after 2 weeks following diet administration, making it a possible good biomarker candidate for early diagnosis of atherosclerosis and aortic valve lesion development. Citrullinated H3R8-nucleosome also represent circulating biomarkers of NETosis. The elevation of these modified histones in response to cholesterol combined with cardiac irradiation may point to new NET-related mechanisms leading to RIHD. Its role as a biomarker of early RIHD also deserves further investigation.

Some biological processes were also specifically triggered by cardiac irradiation in the aorta (e.g., ECM remodeling, platelet degranulation) and aortic valve (e.g., ephrin signaling pathway, Wnt signaling pathway, and glucocorticoid metabolism).

Platelet degranulation was found to be increased in the aorta tissue of rabbit groups fed with a cholesterol-enriched diet, combined either with cardiac irradiation or without cardiac irradiation. This result was corroborated by increased plasma levels of PF4 in both groups. Importantly, PF4 has already been associated with atherosclerosis development and could represent another biomarker of atherosclerosis (43) and possibly of valve remodeling.

## Limitations

Our study has some limitations, including that only male rabbits were included. Also, we did not detect any impact of irradiation by itself on the cardiac structures over the analyzed 8-week period. Longer time protocols should be undertaken to evaluate the sole effect of irradiation, without the influence of any diet. Also, the addition of cardiac irradiation to a lard-enriched diet would be interesting to determine whether irradiation-mediated inflammation would potentiate lard-induced lesions. Furthermore, tissue structure was only assessed by histological analyses, which did not allow for 3D visualization of the lesions, which could provide insights into their cellular and extracellular composition. Finally, RNAseq analysis was performed on complete tissues and therefore identification of specific cellular types was not possible. Single-cell RNA sequencing could bring further information regarding the impact of treatments on tissue-specific cellular type enrichment and cellular process activation or inhibition.

## Conclusion

Our study showed that cholesterol- and lard-enriched diets, and cardiac irradiation combined with a cholesterol-enriched diet induce the development of distinct lesions in the aorta, the aortic valve, and the mitral valve. Lard- and cholesterol-induced valvular and vascular lesions were different both in terms of histological modifications and in the underlying biological process. While cholesterol led to a global increase of inflammation-related processes, lard causes arterial and aortic valve calcification *via* distinct muscle-related mechanisms. Cardiac irradiation enhanced inflammatory processes and calcification in the arteries and aortic valve. At the systemic level, markers of platelet activation, neutrophils, and specific histone modifications in nucleosomes could represent new highly valuable biomarkers for the early diagnosis of ongoing inflammation-related atherosclerosis and heart valve remodeling.

## Data Availability Statement

The datasets presented in this study can be found in online repositories. The names of the repository/repositories and accession number(s) can be found below: https://www.ncbi.nlm.nih.gov/geo/query/acc.cgi?acc=GSE193060.

## Ethics Statement

The animal study was reviewed and approved by Commission Ethique Animale ULiège, Université de Liège, Liège, Belgium.

## Author Contributions

CO, PL, and AN contributed to the conception and design of the study. ZJ performed the statistical analysis. ND and CO wrote the manuscript. CD'E, AH, MH, RD, M-LN, AP, DP, and ND performed the experiments. MH, JT, PDe, and PL contributed to data interpretation. FL, PC, PM, JP, PDe, and PDr performed the experiments of provided tools and expertise for setting up the animal models. All authors contributed to manuscript revision, read, and approved the submitted version.

## Funding

This project was supported by grants from the Région Wallonne (Projet Complément FEDER BIOMED HUB, convention 1510605) and the Fonds Léon Frédéricq (FLF, Prix de la Province de Liège 2019-2020). CO is Research Director at the National Funds for Scientific Research, Belgium (FRS-FNRS). This study was sponsored by Volition SRL. This study received funding from Belgian Volition SRL. The funder was involved in the study design and in the collection, analysis, and interpretation of the data of nucleosome plasma levels.

## Conflict of Interest

MH and DP were employed by Belgian Volition SRL. JT was employed by Volition America. The remaining authors declare that the research was conducted in the absence of any commercial or financial relationships that could be construed as a potential conflict of interest.

## Publisher's Note

All claims expressed in this article are solely those of the authors and do not necessarily represent those of their affiliated organizations, or those of the publisher, the editors and the reviewers. Any product that may be evaluated in this article, or claim that may be made by its manufacturer, is not guaranteed or endorsed by the publisher.

## Abbreviations

Ao: aorta
AoV: aortic valve
α-SMA: α-smooth muscle actin
CAD: coronary artery disease
CAVD: calcific aortic valve disease
CVD: cardiovascular disease
DKK1: Dickkopf WNT Signaling Pathway Inhibitor 1
CHT: cholesterol
CT: computed tomography
Ctrl: control
DEG: differentially expressed genes
ECM: extracellular matrix
GAG: glycosaminoglycan
HDL: high-density lipoprotein
IR: irradiation
LDL: low-density lipoprotein
MV: mitral valve
NET: neutrophil extracellular trap
PF4: platelet factor 4
PGF2α: prostaglandin F2α
RIHD: radiation-induced heart disease
VHD: valvular heart disease
Vit.D2: Vitamin D2
Wnt: Wingless Integration site.
