# Supplementary material for: Differential Biological Effects of Dietary Lipids and Irradiation on the Aorta, Aortic Valve, and the Mitral Valve
Source: Front Cardiovasc Med. 2022 Feb 28;9:839720. doi: 10.3389/fcvm.2022.839720 (PMC8918952; doi:10.3389/fcvm.2022.839720)
Supplement: Supplementary file 1 [file Data_Sheet_1.PDF]

Supplementary data

Table S1. Top 10 biological processes affected by aorta-specific DEGs as compared to aortic valve after cholesterol-rich diet

GO term	Gene frequency	P-value	DEGs
Neutrophil degranulation	11.453% (41/358)	3.08E-10	*FGR;PLAUR;ALOX5;DNASE1L1;TNFRSF1B;GRN;KCNAB2;STK10;STXBP2;FTL;CTSZ;ATP8B4;GSDMD;MAN2B1;TSPAN14;FOLR3;IRAG2;ADGRE5;ARHGAP9;TNFAIP6;ATP8A1;CD93;CFP;CRACR2A;CHI3L1;SDCBP;IQGAP2;DSN1;CDA;HK3;CYBB;B2M;ANPEP;FTH1;HPSE;RHOG;STING1;ATP6V0C;PSAP;PNP;PECAM1*
Inflammatory response	11.331% (80/706)	4.09E-08	*FGR;CX3CL1;NOS2;SELE;ALOX5;BIRC3;TNFRSF1B;GRN;FOXP3;IFI35;PTGS2;IL4R;CST7;TIMP1;ACOD1;CCN4;GSDMD;PIK3CG;AKNA;GCG;FN1;IL1RL1;IL18RAP;CCR2;CXCR4;ADGRE5;TNFAIP6;ADCY4;RHBDF2;THEMIS2;ALOX5AP;CHI3L1;CD180;IRAK2;IL10;THBS1;CXCL9;NLRP12;EPHA2;ECM1;HTR7;MMP3;FCGR1A;BMP6;CD96;GBP5;IL34;SLAMF8;IFNGR2;IL6R;VCAM1;NLRP3;CD200R1;TLR3;CYBB;NOD2;NFKBID;CXCL10;ADRB2;ADORA2B;PTGER4;CXCR6;CERS6;C1QA;XCR1;SELP;METRNL;GBA;CDH5;CSF1R;STING1;USP18;MC2R;APOD;IL1RAP;CASP4;PLCG2;GPSM3;TREX1;S1PR3*
Positive regulation of angiogenesis	14.724% (24/163)	3.09E-07	*CX3CL1;GRN;PTGS2;NRP1;PPP1R16B;SRPX2;FLT1;NR2E1;CXCR4;CHI3L1;IL10;THBS1;ECM1;ADAM12;RUNX1;HK2;S100A1;TLR3;NOS3;CYBB;PRKCB;CX3CR1;CDH5;PIK3R6*
Interferon-gamma-mediated signaling pathway	24.138% (14/58)	8.00E-07	*OAS1;OAS3;OAS2;TRIM38;PARP9;NLRC5;IRF8;FCGR1A;IFNGR2;VCAM1;B2M;PARP14;IRF7;HLA-DPB1*
Platelet degranulation	15.842% (16/101)	1.33E-06	*CD9;STXBP2;PDGFB;TIMP1;LGALS3BP;VWF;FN1;ABCC4;THBS1;ECM1;CYRIB;SELP;PSAP;F5;ITGB3;PECAM1*
Positive regulation of cell migration	10.284% (47/457)	7.08E-06	*FGR;CX3CL1;SELE;GRN;PTGS2;GTSE1;EDN1;SEMA5B;CASS4;LAMB1;NRP1;PDGFB;LGMN;SRPX2;FLT1;CEMIP;CCN4;PIK3CG;VTN;FN1;CD274;CCR2;CXCR4;TNFAIP6;PREX1;ATP8A1;P2RX4;SDCBP;THBS1;LEF1;PAK1;IL34;IL6R;NOS3;CX3CR1;CXCL10;HAS2;SELP;CDH5;CSF1R;SHTN1;SEMA4D;SEMA4A;PLCG2;GPSM3;ITGB3;PECAM1*
Immune response	10.707% (168/1569)	7.16E-06	*FGR;CX3CL1;NOS2;PLAUR;ALOX5;DNASE1L1;WAS;MARCO;BIRC3;TNFRSF1B;POU2F2;GRN;PARP3;FOXP3;IFI35;BCL3;KCNAB2;STK10;PAG1;STXBP2;IL4R;CST7;EDN1;FTL;OAS1;NFATC2;CTSZ;BMX;ACOD1;ATP8B4;GSDMD;FGL1;MAN2B1;DNASE2;PIK3CG;TRIM14;DDX58;TSPAN14;ICAM2;DHX58;VTN;CLNK;FOLR3;SLC15A3;ARPC3;OAS3;OAS2;TRIM38;GCG;IL1RL1;IL18RAP;RAB29;IRAG2;CD274;TASL;CCR2;TNFSF10;CXCR4;ADGRE5;ARHGAP9;TNFAIP6;ATP8A1;CD93;CFP;ADCY4;RHBDF2;CRACR2A;ARPC1B;THEMIS2;XAF1;CHI3L1;CD180;RSAD2;CTSL;DOCK10;IL10;SDCBP;THBS1;IFI44L;IFI44;PARP9;CXCL9;LEF1;NLRC5;IRF8;IFNAR1;ECM1;GZMA;IQGAP2;TNFRSF21;HTR7;PAK1;DSN1;FCGR1A;BMP6;CD96;CYRIB;GBP5;SAMSN1;VSIG4;IL34;MX1;SLAMF8;CDA;IFNGR2;RUNX1;C1R;IL6R;HK3;VCAM1;NLRP3;TRIM58;SLC15A2;CD200R1;DTX3L;TLR3;CYBB;PRKCB;CLEC4E;B2M;ANPEP;NOD2;NFKBID;FTH1;CX3CR1;TAP1;INPP5D;CXCL10;ADRB2;ADORA2B;PTGER4;CXCR6;HPSE;PARP14;C1QB;C1QA;XCR1;ZNF683;RHOG;SHMT2;C1S;FES;CSF1R;STING1;USP18;MC2R;IRF7;ATP6V0C;ISG15;SEMA4D;IL1RAP;SEMA4A;DAPK1;CASP4;PSAP;PLCG2;MBP;PNP;PLPP4;IFIT1B;IGHM;GPSM3;TREX1;HLA-DPB1;PSMB9;PECAM1;PIK3R6;RAB7B*
Response to lipopolysaccharide	12.741% (33/259)	1.38E-05	*CX3CL1;NOS2;SELE;TNFRSF1B;FOXP3;PTGS2;EDN1;CSF2RB;ACOD1;IL10RA;MAPKAPK3;CD274;CD180;IRAK2;IL10;CXCL9;IRF8;IFNAR1;ANKRD1;BMP6;CD96;VCAM1;NLRP3;NOS3;NOD2;CX3CR1;CXCL10;PTGER4;SELP;PENK;LITAF;NUGGC;PLCG2*

Negative regulation of type I interferon production	26.667% (8/30)	1.64E-05	*ACOD1;DDX58;DHX58;IL10;NLRC5;UBA7;TRAIP;ISG15*
Negative regulation of type I interferon-mediated signaling pathway	35.294% (6/17)	2.64E-05	*OAS1;OAS3;NLRC5;USP18;ISG15;TREX1*

Table S2. Top 10 biological processes affected by aortic valve-specific DEGs as compared to aorta after cholesterol-rich diet

GO term	Gene frequency	P-value	DEGs
Muscle filament sliding	33.333% (9/27)	1.26E-12	*ACTN2;MYH7;MYL2;TNNC1;MYBPC3;ACTA1;ACTC1;MYL3;DES*
Sarcomere organization	25% (8/32)	2.29E-09	*MYOM2;ACTN2;LDB3;CSRP3;MYPN;MYLK3;LMOD2;MYOZ2*
Cardiac muscle contraction	9.821% (11/112)	6.73E-09	*MYH7;TNNC1;RGS2;CASQ2;CSRP3;MYBPC3;RNF207;ACTC1;MYL3;NKX2-5;RYR2*
Ventricular cardiac muscle tissue morphogenesis	20.588% (7/34)	1.86E-08	*MYH7;MYL2;TNNC1;MYBPC3;MYL3;NKX2-5;RYR2*
Cardiac myofibril assembly	31.25% (5/16)	2.28E-07	*MYL2;CSRP3;MYLK3;ACTC1;NRAP*
Regulation of the force of heart contraction	25% (5/20)	7.86E-07	*MYH7;MYL2;CSRP3;MYL3;RYR2*
Muscle contraction	8.503% (25/294)	2.88E-06	*MYOM2;ACTN2;MYH7;HTR2A;MYL2;TNNC1;RGS2;CASQ2;MYOT;CSRP3;CKMT2;MYBPC3;CHRND;MYOM3;ACTA1;RNF207;ACTC1;MYL3;PGAM2;LMOD2;DES;TRIM72;NKX2-5;MB;RYR2*
Creatine metabolic process	60% (3/5)	7.19E-06	*CKM;CKMT2;CKB*
Regulation of striated muscle contraction	10.976% (9/82)	1.24E-05	*MYH7;MYL2;RGS2;CASQ2;MYBPC3;RNF207;MYL3;NKX2-5;RYR2*
Negative regulation of myotube differentiation	23.077% (3/13)	0.000195	*CCN3;TRIM72;NKX2-5*

Table S3. Top 10 biological processes affected by common aorta and aortic valve DEGs after cholesterol-rich diet

GO term	Gene frequency	P-value	DEGs
Neutrophil degranulation	12.849% (46/358)	2.56E-16	*ITGAL;TYROBP;PTPRC;CPPED1;PYCARD;CTSH;CTSC;BIN2;PTPN6;PADI2;NPC2;PLAU;HVCN1;NCKAP1L;CD68;GMFG;LGALS3;CHIT1;TLR2;PRCP;GGH;GLIPR1;ITGAX;CD53;PLAC8;DOK3;FCER1G;CSTB;ITGB2;FCGR3B;CTSS;FABP5;CTSB;SYK;CLEC4D;PTAFR;ITGAM;GUSB;GLB1;CD14;C3AR1;OLR1;IGF2R;C5AR1;ORM2;CLEC5A*
Antigen processing and presentation of exogenous peptide antigen via MHC class II	22.785% (18/79)	2.32E-12	*CD74;FCGR2B;KIF4A;CTSV;KIF23;KIF11;CENPE;KIF2C;FCER1G;RACGAP1;CTSS;HLA-DQB1;HLA-DRB1;CTSE;HLA-DOA;HLA-DMA;HLA-DRA;HLA-DMB*
Positive regulation of tumor necrosis factor production	20.988% (17/81)	3.91E-11	*TYROBP;PTPRC;PYCARD;CCR2;TLR4;TLR2;SYK;PTAFR;CD14;CLEC7A;RASGRP1;CCL19;TLR1;LILRA5;SPN;ORM2;CCL3*
Lipopolysaccharide-mediated signaling pathway	27.907% (12/43)	9.73E-10	*HCK;CCL2;LY86;PTPN22;TLR4;TLR2;SCIMP;PTAFR;CD14;TNF;LYN;CCL3*
Mitotic spindle organization	16.168% (27/167)	1.04E-09	*NDC80;AURKA;TPX2;KIF4A;MYBL2;TTK;CENPA;NEK2;CENPK;DLGAP5;SGO1;CCNB1;CDCA8;KIF23;KNL1;KIF11;CENPE;KIF2C;NUF2;BUB1B;RACGAP1;BUB1;AURKB;KNTC1;ERCC6L;PRC1;KIFC1*
Positive regulation of interleukin-6 production	20% (15/75)	1.15E-09	*TYROBP;CD74;TLR8;PYCARD;IL1RL2;TLR4;TLR2;SCIMP;SYK;PTAFR;CLEC7A;TLR1;LILRA5;AIF1;TNF*
Inflammatory response	9.348% (66/706)	5.58E-09	*ITGAL;BTK;TYROBP;NR1H3;GLP2R;FCGR2B;PTPRC;BLNK;TREM2;HMOX1;ABHD12;HCK;TLR8;PYCARD;PLD3;LIPA;CCL7;CCL2;CTSC;LPCAT3;LY86;HRH2;IL1RL2;SPP1;CCR2;ADGRE2;IRF5;CD68;LDLR;AOAH;IL1RN;TLR4;TLR2;PRCP;CASP1;KLRG1;PLA2G7;PIK3AP1;ITGB2;REL;TNFAIP8L2;CCR1;SYK;PTAFR;ITGAM;CD14;C3AR1;CLEC7A;RASGRP1;CCL19;OLR1;NRROS;TLR1;SPHK1;CD28;LILRA5;HLA-DRB1;C5AR1;AIF1;CD200R1L;ORM2;TNF;IRGM;IL10RB;LYN;CCL3*
Cellular defense response	31.25% (10/32)	6.89E-09	*TYROBP;IL1RL2;NCF2;PTK2B;CCR2;KLRG1;CCR9;C5AR1;SPN;CLEC5A*
Positive regulation of interleukin-1 beta production	24.444% (11/45)	2.12E-08	*TYROBP;TLR8;PYCARD;TLR4;CASP1;CLEC7A;CCL19;LILRA5;ORM2;TNF;CCL3*
Positive regulation of ERK1 and ERK2 cascade	11.834% (20/169)	2.92E-08	*CD74;ATP6AP1;PTPRC;TREM2;PYCARD;CCL7;CCL2;PTK2B;PTPN22;GPNMB;TLR4;SCIMP;CCR1;GPR183;RASGRP1;CCL19;HLA-DRB1;C5AR1;TNF;CCL3*

Table S4. Top 10 biological processes affected by aortic valve-specific DEGs as compared to mitral valve after cholesterol-rich diet

GO term	Gene frequency	P-value	DEGs
Cell division	9.524% (50/525)	1.63E-10	*KLHL13;ANLN;SPAG5;NDC80;AURKA;TPX2;KIF4A;CCNE1;NCAPG;TIMELESS;KIF20A;ECT2;ACTR3;CENPA;NEK2;KIF14;CKS2;KNSTRN;E2F8;SGO1;TOP2A;CCNB1;CKAP2;NUSAP1;KIF23;KNL1;KIF11;CEP55;KIF20B;CENPE;PLK4;KIF2C;NUF2;SFRP2;PDGFC;CCNB2;CDC25C;RACGAP1;BUB1;CDK1;UBE2C;CCNE2;AURKB;MACC1;KNTC1;CDCA2;EVI2B;ERCC6L;PRC1;KIFC1*
Antigen processing and presentation of exogenous peptide antigen via MHC class II	17.722% (14/79)	1.61E-09	*CD74;FCGR2B;KIF4A;CTSV;KIF23;KIF11;CENPE;KIF2C;RACGAP1;HLA-DQB1;AP1S2;HLA-DRB1;HLA-DOA;HLA-DRA*
Muscle filament sliding	33.333% (9/27)	3.59E-09	*ACTN2;MYH7;MYL2;TNNC1;MYBPC3;ACTA1;ACTC1;MYL3;DES*
Mitotic spindle organization	14.371% (24/167)	3.69E-09	*NDC80;AURKA;TPX2;KIF4A;TTK;CENPA;NEK2;CENPK;DLGAP5;SGO1;CCNB1;KIF23;KNL1;KIF11;CENPE;KIF2C;NUF2;RACGAP1;BUB1;AURKB;KNTC1;ERCC6L;PRC1;KIFC1*
Regulation of attachment of spindle microtubules to kinetochore	53.846% (7/13)	9.85E-08	*SPAG5;ECT2;NEK2;KNSTRN;CCNB1;RACGAP1;AURKB*
Mitotic spindle midzone assembly	71.429% (5/7)	1.02E-07	*KIF4A;KIF23;RACGAP1;AURKB;PRC1*
Sarcomere organization	25% (8/32)	1.06E-06	*MYOM2;ACTN2;LDB3;CSRP3;MYPN;MYLK3;LMOD2;MYOZ2*
Cardiac muscle contraction	8.929% (10/112)	1.55E-06	*MYH7;TNNC1;CASQ2;CSRP3;MYBPC3;RNF207;ACTC1;MYL3;NKX2-5;RYR2*
Protein localization to kinetochore	33.333% (6/18)	1.62E-06	*NDC80;TTK;KNL1;CDK1;HASPIN;AURKB*
mitotic cytokinesis	17.46% (11/63)	2.40E-06	*ANLN;KIF4A;KIF20A;ECT2;CENPA;CKAP2;NUSAP1;KIF23;CEP55;KIF20B;RACGAP1*

Table S5. Top 10 biological processes affected by mitral valve-specific DEGs as compared to aortic valve after cholesterol-rich diet

GO term	Gene frequency	P-value	DEGs
Cholesterol biosynthetic process	7.547% (4/53)	1.13E-05	*IDI1;FDFT1;HMGCS1;HMGCR*
Farnesyl diphosphate metabolic process	40% (2/5)	6.89E-05	*FDFT1;HMGCS1*
Folic acid transport	33.333% (2/6)	0.000103	*SLC46A1;FOLR3*
Isoprenoid biosynthetic process	12% (3/25)	0.000424	*IDI1;HMGCS1;HMGCR*
Regulation of lipid metabolic process	1.775% (6/338)	0.001076	*IDI1;FDFT1;HMGCS1;HMGCR;PIK3R5;APOD*
Phospholipid biosynthetic process	1.288% (3/233)	0.004227	*IDI1;HMGCS1;PIK3R5*
Drug transport	22.222% (2/9)	0.005181	*SLC46A1;ABCC3*
Regulation of muscle organ development	1.695% (2/118)	0.005225	*HMGCR;TIFAB*

Table S6. Top 10 biological processes affected by common aortic and mitral valve DEGs after the cholesterol-rich diet

GO term	Gene frequency	P-value	DEGs
Neutrophil degranulation	9.777% (35/358)	1.01E-16	*ITGAL;TYROBP;PTPRC;PYCARD;CTSH;BIN2;PTPN6;PADI2;PLAU;NCKAP1L;CD68;GMFG;CHIT1;GGH;GLIPR1;ITGAX;DOK3;FCER1G;ITGB2;FCGR3B;CTSS;FABP5;CTSB;SYK;CLEC4D;PTAFR;ITGAM;GUSB;GLB1;CD14;OSCAR;C3AR1;OLR1;IGF2R;C5AR1*
Inflammatory response	6.091% (43/706)	2.26E-10	*ITGAL;BTK;TYROBP;HGF;NR1H3;GLP2R;PTPRC;BLNK;TREM2;ABHD12;HCK;TLR8;PYCARD;PLD3;LIPA;LY86;SPP1;GPR68;CCR2;IRF5;CD68;LDLR;AOAH;IL1RN;CASP1;KLRG1;PIK3AP1;ITGB2;TNFAIP8L2;CCR1;SYK;PTAFR;ITGAM;CD14;C3AR1;CLEC7A;OLR1;NRROS;CD28;C5AR1;AIF1;IRGM;IL10RB*
Phagocytosis. engulfment	21.739% (10/46)	1.23E-08	*MSR1;TREM2;BIN2;NCKAP1L;RAC2;ITGB2;ARHGAP25;ABCA1;ITGAM;AIF1*
Integrin-mediated signaling pathway	11.765% (12/102)	1.83E-08	*ITGAL;FYB1;HCK;ITGA4;PLEK;PTK2B;ITGAX;VAV1;ITGAD;ITGB2;SYK;ITGAM*
B cell receptor signaling pathway	20% (10/50)	1.04E-07	*BTK;CD22;PTPRC;BLNK;LPXN;PTPN6;NCKAP1L;PTPN22;SYK;KLHL6*
Regulation of neutrophil degranulation	80% (4/5)	1.86E-07	*ITGB2;SYK;PTAFR;ITGAM*
Response to molecule of fungal origin	80% (4/5)	1.86E-07	*BTK;SCIMP;SYK;CLEC7A*
Cellular defense response	21.875% (7/32)	2.39E-07	*TYROBP;NCF2;PTK2B;CCR2;KLRG1;C5AR1;SPN*
Positive regulation of interleukin-10 production	21.212% (7/33)	3.00E-07	*HGF;TREM2;PYCARD;CD83;SYK;CLEC7A;CD28*
Innate immune response	6.21% (39/628)	6.72E-07	*BTK;TYROBP;NR1H3;CD84;TREM2;HCK;TLR8;CORO1A;PYCARD;PTPN6;LY86;KYNU;NCF2;PTK2B;IRF5;PTPN22;OASL;DDX60;CASP1;KLRG1;VAV1;MCOLN2;SLA;FCER1G;SLAMF6;TNFAIP8L2;DEFB1;SYK;CLEC4D;PTAFR;ITGAM;CD14;CLEC7A;CADM1;SIGLEC15;MPEG1;AIF1;IRGM;MMP12*

Table S7. Top 10 biological processes affected by aorta DEGs after lard-rich diet

GO term	Gene frequency	P-value	DEGs
Extracellular matrix organization	5.882% (20/340)	5.28E-08	*IBSP;TNC;COL23A1;COL11A1;MMP9;SPP1;LCP1;ITGAX;COL8A1;ADAM12;ITGAD;ACAN;RUNX1;ITGB2;MATN1;MELTF;ITGAM;COL24A1;COL11A2;MMP12*
Peptide antigen assembly with MHC protein complex	60% (3/5)	1.21E-06	*HLA-DRB1;HLA-DRA;HLA-DMB*
Ossification	4.217% (14/332)	3.52E-06	*IBSP;TNC;CHRDL2;COL11A1;MMP9;SPP1;EGR2;RUNX2;RSPO2;RUNX1;MATN1;CLEC3A;COL11A2;CLEC5A*
Collagen fibril organization	7.229% (6/83)	3.59E-06	*COL23A1;COL11A1;COL8A1;ACAN;COL24A1;COL11A2*
Positive regulation of angiogenesis	4.294% (7/163)	2.21E-05	*CHI3L1;ITGAX;ADAM12;RUNX1;ITGB2;S100A1;SPHK1*
Osteoblast differentiation	3.889% (7/180)	2.33E-05	*IBSP;TNC;SPP1;RUNX2;RSPO2;COL11A2;CLEC5A*
Endodermal cell differentiation	11.429% (4/35)	2.73E-05	*COL11A1;MMP9;COL8A1;ITGB2*
Antigen processing and presentation of exogenous peptide antigen via MHC class II	6.329% (5/79)	4.68E-05	*CD74;FCER1G;HLA-DRB1;HLA-DRA;HLA-DMB*
Dendritic cell antigen processing and presentation	21.429% (3/14)	7.25E-05	*CD74;HLA-DRB1;HLA-DRA*
Positive regulation of superoxide anion generation	17.647% (3/17)	7.87E-05	*ITGB2;ITGAM;CLEC7A*

Table S8. Top 10 biological processes affected by aortic valve DEGs after lard-rich diet

GO term	Gene frequency	P-value	DEGs
Muscle filament sliding	29.63% (8/27)	2.33E-15	*ACTN2;MYH7;MYL2;TNNC1;MYBPC3;ACTA1;ACTC1;MYL3*
Sarcomere organization	21.875% (7/32)	1.64E-12	*MYOM2;ACTN2;LDB3;CSRP3;MYPN;ANKRD1;LMOD2*
Cardiac muscle contraction	8.036% (9/112)	2.44E-12	*MYH7;TNNC1;CASQ2;CSRP3;MYBPC3;RNF207;ACTC1;MYL3;RYR2*
Ventricular cardiac muscle tissue morphogenesis	17.647% (6/34)	2.93E-10	*MYH7;MYL2;TNNC1;MYBPC3;MYL3;RYR2*
Regulation of the force of heart contraction	25% (5/20)	1.51E-09	*MYH7;MYL2;CSRP3;MYL3;RYR2*
Cardiac myofibril assembly	25% (4/16)	7.56E-08	*MYL2;CSRP3;ACTC1;NRAP*
Regulation of striated muscle contraction	8.537% (7/82)	2.42E-07	*MYH7;MYL2;CASQ2;MYBPC3;RNF207;MYL3;RYR2*
Regulation of actin filament-based movement	10% (4/40)	1.85E-05	*TNNC1;MYBPC3;RNF207;RYR2*
Skeletal muscle thin filament assembly	40% (2/5)	6.89E-05	*ACTA1;ACTC1*
Mesenchyme migration	40% (2/5)	6.89E-05	*ACTA1;ACTC1*

Table S9. Top 10 biological processes affected by aorta-specific DEGs as compared to aortic valve after cholesterol-rich diet combined with irradiation

GO term	Gene frequency	P-value	DEGs
Inflammatory response	10.907% (77/706)	1.71E-09	*CX3CL1;NOS2;STAB1;ALOX5;TNFRSF1B;STAP1;TNFRSF1A;IFI35;IL4R;LXN;SIGLEC1;ICAM1;GGT5;MYH9;BDKRB1;CD40;HAMP;NOD1;AKNA;GCG;TNFAIP3;VAMP8;CLU;ADCY7;ADGRE5;PTGER2;ADCY4;LBP;CHI3L1;RXFP2;IRAK2;TSPAN2;CCL21;ANKRD42;THBS1;FURIN;IGFBP4;SIGLEC10;EPHA2;XCL1;ECM1;PLK2;CRHBP;TIRAP;BMP6;CD96;IL34;NLRX1;VCAM1;DNASE1L3;ZC3H12A;F2RL1;HTR4;TLR3;JAM3;NFKBID;ADRB2;ADORA2B;FPR1;CXCR6;MYD88;CYSLTR1;C1QA;XCR1;SELP;CMKLR1;NPPA;CD163;CDH5;GPBAR1;CSF1;STING1;CXCR3;APOD;SRC;C4B;ADORA3*
Extracellular matrix organization	15% (51/340)	2.42E-07	*ITGA3;TNFRSF1B;TNC;COL23A1;COL11A1;TIE1;TNFRSF1A;BCL3;ITGA8;COL5A3;MMP2;ICAM1;LAMB1;ITGA6;CRISPLD2;ENG;ICAM2;COL1A1;VWF;COL12A1;NR2E1;LAMA4;POSTN;MYH11;LOXL2;CTSL;THBS1;LOXL4;ITGB7;FBLN5;FURIN;COL8A1;ADAMTS16;P4HA3;DST;CREB3L1;ACAN;ITGA5;VCAM1;ADAMTS9;JAM3;SPINT1;HAS2;COL24A1;ANGPTL7;A2M;ADAMTSL1;SULF2;COLQ;ITGB3;PECAM1*
Cellular response to tumor necrosis factor	10.504% (25/238)	1.38E-06	*CX3CL1;TNFRSF1B;TNFRSF1A;EDN1;ICAM1;CD40;HAMP;COL1A1;TNFAIP3;PLVAP;CHI3L1;POSTN;CCL21;THBS1;XCL1;CRHBP;TNFRSF21;ST18;ANKRD1;TNFSF13;VCAM1;CLDN1;ZC3H12A;F2RL1;HAS2*
Positive regulation of angiogenesis	14.11% (23/163)	7.76E-06	*CX3CL1;TIE1;CD40;FLT1;ENG;NR2E1;VEGFA;PGF;CHI3L1;THBS1;ECM1;PLK2;S100A1;ITGA5;ZC3H12A;TLR3;BMPER;NOS3;PRKCB;ANGPTL4;CDH5;CXCR3;GDF2*
Regulation of cell adhesion	11.282% (66/585)	8.42E-06	*PLXND1;ITGA3;CX3CL1;ALOX5;VCL;TNC;PPP1R12A;ROCK1;LIMS2;FERMT2;NUAK1;PLXNA2;IL4R;ICAM1;ITGA6;PPM1F;IL27RA;AKNA;ENG;VSIR;ZMIZ1;CYTH1;COL1A1;SH2B3;NEDD9;DUSP22;VEGFA;LAMA4;ZAP70;RREB1;LIF;RARA;POSTN;SERPINE2;HLX;CCL21;LRRC32;THBS1;LEF1;TPM1;PML;MINK1;EPHA2;XCL1;ITPKB;COL8A1;TNFRSF21;PAK1;BMP6;NRG1;VCAM1;ZC3H12A;EMCN;JAM3;NFKBID;HAS2;PEAK1;SELP;FES;IRAK1;CSF1;CXCR3;SPRY4;APOD;SRC;ADGRG1*
Regulation of chemokine (C-X-C motif) ligand 2 production	40% (6/15)	2.47E-05	*OAS1;OAS3;POSTN;TIRAP;F2RL1;MYD88*
Positive regulation of apoptotic process involved in morphogenesis	80% (4/5)	2.49E-05	*TNFRSF1B;TNFRSF1A;BAX;PML*
Platelet degranulation	14.851% (15/101)	8.59E-05	*VCL;LGALS3BP;VWF;VEGFA;CLU;THBS1;ECM1;LHFPL2;ALB;PCDH7;SELP;A2M;F5;ITGB3;PECAM1*
Regulation of immune response	10.789% (82/760)	9.73E-05	*TNFRSF1B;STAP1;PARP3;IFI35;IL4R;BAX;OAS1;ICAM1;GGT5;MYH9;CD40;NFATC2;BMX;KCNN4;IL27RA;VSIR;ICAM2;DHX58;COL1A1;OAS3;TREML2;ZAP70;GCG;CR2;TNFAIP3;VAMP8;CLU;ADCY7;IL13RA2;PTGER2;PLD2;ADCY4;LBP;RFTN1;RARA;RXFP2;PRAM1;HLX;SLC15A4;ITGB7;FURIN;NLRC5;CMTM3;XCL1;ECM1;PLK2;TNFRSF21;DGKZ;PAK1;TIRAP;CD96;C1R;NLRX1;SH2B2;CD300LG;TNFSF13;VCAM1;ZC3H12A;F2RL1;HTR4;TLR3;BTNL9;PRKCB;NFKBID;ADRB2;ADORA2B;FPR1;CYSLTR1;C1QA;CMKLR1;NPPA;A2M;CD163;GPBAR1;C1S;FES;STING1;PTPN1;SRC;PLPP4;C4B;CD8B2*
Collagen fibril organization	15.663% (13/83)	0.000145	*COL23A1;COL11A1;COL5A3;ITGA6;COL1A1;COL12A1;LOXL2;LOXL4;COL8A1;P4HA3;DST;ACAN;COL24A1*

Table S10. Top 10 biological processes affected by aortic valve-specific DEGs as compared to aorta after cholesterol-rich diet combined with irradiation

GO term	Gene frequency	P-value	DEGs
Heart development	20.998% (101/481)	1.16E-05	*CACNA1G;HDAC9;CC2D2A;ADAMTS6;ERBB3;TGFBR3;GLI2;TEAD2;ACACB;ACTN2;NEBL;MEF2C;KCNK2;GRHL2;DSP;HIF1A;MTHFD1;NFATC4;STK4;FOXF1;EYA1;PTN;GLI3;BMPR1A;SOX6;MYL2;CDKN1B;RBM24;BVES;TBX18;HAND1;TFDP2;WNT5A;TNNC1;ID2;RGS2;RGS4;ID3;PROX1;NRP2;PKD2;POPDC2;GJB6;LDB3;OBSL1;SOX9;BMP2;FLRT3;SIX1;SMO;RAMP2;PPARG;POPDC3;PCNA;MYO18B;MBD2;PDGFRA;AHI1;ALPK3;YAP1;IFT172;PLCE1;COX17;MYLK3;GREB1L;ERBB2;SYPL2;ANK2;SFRP2;TENM4;FREM2;ABI3BP;MMP21;PDLIM3;DRC1;MYL3;VANGL2;XIRP2;IFT122;DLC1;TMEM100;SYNPO2L;GREM1;AXIN2;EFNA1;PTK2;ROBO1;ZFPM2;HSPB7;CAV3;GJC1;NKX2-5;RIPPLY3;PDE2A;DYNC2H1;EPHB4;MB;ALPK2;MIR222;LEFTY1;DNAAF4*
Ephrin receptor signaling pathway	29.167% (21/72)	5.59E-05	*FYN;RHOA;ACTB;PAK3;EPHB6;ACTR3;EPHA4;EPHB2;EPHA7;ACTR2;PSEN2;EPHA5;EPHA1;AP2M1;NCSTN;EFNA1;PTK2;SDC2;ANKS1B;EPHB4;ARPC4*
Axon guidance	24.896% (60/241)	6.43E-05	*FYN;SEMA3B;LAMA3;NGFR;SPTB;GLI2;RAP1GAP;IGSF9;LAMA1;HOXA2;GLI3;LGI1;SEMA5A;WNT5A;SPTBN1;EPHA4;NRP2;MYOT;PLCG1;FLRT3;SMO;LGR6;EPHB2;CHL1;EPHA7;SEMA6D;ERBB2;SEMA6C;BOC;EPHA5;EPHA1;NFIB;NCAM1;SEMA3D;KIF5A;SPTBN4;NTN3;ZSWIM5;PLXNB1;TTC8;EFNA1;PTK2;ROBO1;ALCAM;B3GNT2;FEZ2;GAP43;LAMB2;PTPRM;BDNF;GRB2;NRXN1;UNC5C;DCC;RELN;EPHB4;LAMA2;SPTAN1;PLXNB3;L1CAM*
Response to mechanical stimulus	19.337% (35/181)	7.11E-05	*FYN;DCN;ATP1A2;RHOA;KCNK2;ANGPT2;HTR2A;HPN;PTN;P2RX3;STAT1;PKD2;PPL;SOX9;IL1B;GDF5;KCNA5;PPARG;ATP8A2;MBD2;MEIS2;ACTA1;DRD2;ANKRD23;ATP1A1;ITGA2;SOST;PTK2;JUP;CHRNA9;NRXN1;CAV3;BACE1;PDE2A;BGLAP*
Cochlea morphogenesis	47.368% (9/19)	0.000132	*MYO3B;GLI2;EYA1;HPN;TBX18;WNT5A;SOX9;SIX1;FRZB*
Glucocorticoid metabolic process	31.579% (6/19)	0.000192	*APOA1;BMP2;YWHAH;STAR;ATP1A1;MECP2*
Negative regulation of canonical Wnt signaling pathway	22.297% (33/148)	0.000206	*HECW1;PTPRU;JADE1;PSME1;PSMA6;PSME2;STK4;GLI3;TBX18;WNT5A;PSMD14;SOX9;BMP2;DAB2IP;NKD1;PSMA5;SOX13;RBMS3;SFRP2;DKK2;PSMB4;FRZB;AMER2;GREM1;SOST;AXIN2;MCC;MLLT3;FZD4;PSMD2;NKX2-5;AMER1;MIR29B2*
Calcium ion-regulated exocytosis of neurotransmitter	53.846% (7/13)	0.000282	*SYT1;STX1B;RIMS3;SYT6;STXBP1;RIMS2;DOC2B*
Regulation of dendritic spine morphogenesis	37.5% (15/40)	0.000328	*ARHGAP44;PAK3;LZTS3;EEF2K;CAPRIN2;CUX2;EPHA4;ACTR2;PPP1R9A;EFNA1;TANC2;CFL1;LRRK2;RELN;SRCIN1*
Negative regulation of epithelial cell proliferation	20.611% (27/131)	0.000459	*NGFR;TGFBR3;RAP1GAP;MEF2C;HPN;PTN;CDKN1B;WNT5A;STAT1;KLF9;SOX9;GDF5;PPARG;DAB2IP;IFT172;EAF2;SFRP2;NFIB;CDKN2B;XDH;COL4A3;ROBO1;WNT10B;MCC;PTPRM;MIR222;MIR29B2*

Table S11. Top 10 biological processes affected by common aortic valve and aorta DEGs after cholesterol-rich diet combined with irradiation

GO term	Gene frequency	P-value	DEGs
Neutrophil degranulation	26.816% (96/358)	5.08E-28	*FGR;FUCA2;ITGAL;MGST1;PLAUR;TYROBP;DNASE1L1;DERA;CD44;GRN;CTSA;KCNAB2;STK10;STXBP2;PTPRC;FTL;LYZ;ARSA;MMP9;CTSZ;COTL1;CPPED1;PYCARD;CTSH;ATP8B4;GSDMD;ASAH1;MAN2B1;TSPAN14;CTSC;FOLR3;BIN2;LTA4H;PTPN6;PADI2;IRAG2;NPC2;CAT;PLAU;HVCN1;ARHGAP9;NCKAP1L;TNFAIP6;RAP2C;ATP8A1;CFP;CD68;CRACR2A;GMFG;LGALS3;CHIT1;CD36;TLR2;PRCP;GGH;SDCBP;GLIPR1;ITGAX;CD53;S100A8;PLAC8;IQGAP2;DOK3;ADAM8;CDA;FCER1G;CSTB;ITGB2;HK3;FCGR3B;CTSS;PTX3;FABP5;CTSB;SYK;CYBB;CLEC4D;B2M;FTH1;PTAFR;ITGAM;GUSB;GLB1;CD14;OSCAR;C3AR1;HPSE;OLR1;RHOG;ATP6V0C;LAMP1;IGF2R;C5AR1;PSAP;PNP;CLEC5A*
Inflammatory response	20.963% (148/706)	1.03E-14	*FGR;ITGAL;SELE;BTK;TYROBP;ISL1;BIRC3;NR1H3;CD44;IFNGR1;GRN;FOXP3;TNIP3;GLP2R;FCGR2B;CST7;PTPRC;LYZ;BLNK;TREM2;HMOX1;MMP9;ABHD12;HCK;TLR8;TIMP1;ACOD1;PYCARD;CCN4;GSDMD;PLD3;PIK3CG;LIPA;CCL7;CCL2;CTSC;LPCAT3;CCR6;LY86;HRH2;FN1;IL1RL2;IL1RL1;IL18R1;IL18RAP;SLAMF1;SPP1;GPR68;CCR2;CXCR4;TNFAIP6;ADGRE2;IRF5;CSRP3;CD68;RHBDF2;LDLR;THEMIS2;ALOX5AP;CD180;IL2RA;C5AR2;CD36;NT5E;AOAH;IL10;IL1RN;TLR4;CCN3;TLR2;PRCP;CASP1;CXCL9;KLRG1;NLRP12;DUSP10;S100A8;PLA2G7;EGFR;TRIM55;HTR7;MS4A2;MMP3;FCGR1A;ADAM8;GBP5;PIK3AP1;SLAMF8;IFNGR2;ITGB2;IL6R;NLRP3;REL;TNFAIP8L2;CD200R1;PTX3;CCR1;DAGLB;SYK;CYBB;NOD2;CXCL10;PTAFR;CXCL8;ITGAM;CD14;PTGER4;PIK3CD;C3AR1;CLEC7A;CERS6;RASGRP1;CCL19;ADCY5;OLR1;SAA1;NRROS;TLR1;SPHK1;METRNL;GBA;CD28;CIITA;LACC1;CSF1R;USP18;MC2R;KRT16;LILRA5;IL1RAP;HLA-DRB1;CASP4;C5AR1;PLCG2;AIF1;CD200R1L;MIR204;GPSM3;TREX1;S1PR3;TNF;GPX1;IRGM;IL10RB;CEBPA;LYN;CCL5;CCL3*
Innate immune response	22.771% (143/628)	5.64E-14	*FGR;BTK;TYROBP;WAS;MARCO;CD74;BIRC3;NR1H3;CD44;SLAMF7;IFNGR1;GRN;PRDM1;CD84;FCGR2B;TREM2;POLR3H;COCH;HCK;TLR8;ACOD1;CORO1A;PYCARD;GSDMD;PIK3CG;TRIM14;DDX58;CCL7;CCL2;CLNK;SLC15A3;OAS2;PTPN6;CLEC4A;LY86;IL1RL2;IL18RAP;KYNU;NCF2;SLAMF1;GBP1;IFIT3;TASL;PTK2B;CD244;CFP;IRF5;LGALS3;JCHAIN;XAF1;CD180;PTPN22;RSAD2;OASL;TLR4;IRF4;TLR2;DDX60;CASP1;PARP9;KLRG1;IRF8;VAV1;IFNAR1;DUSP10;S100A8;FCGR1A;ADAM8;MCOLN2;GBP5;VSIG4;SLA;SLAMF8;FCER1G;IFNGR2;C1QC;NLRP3;SLAMF6;REL;TNFAIP8L2;PTX3;DTX3L;DEFB1;SYK;CYBB;C2;CLEC4E;CLEC4D;B2M;NOD2;CX3CR1;CXCL10;PTAFR;RNASE6;ITGAM;CD14;PIK3CD;MALT1;CLEC7A;RASGRP1;CCL19;PARP14;SAA1;TLR1;ZNF683;HLA-DQB1;CIITA;LACC1;SHMT2;CSF1R;LCK;CADM1;USP18;IRF7;LAMP1;KRT16;LILRA5;ISG15;IL1RAP;HLA-DRB1;DAPK1;CASP4;SIGLEC15;MPEG1;PLCG2;IFIT1B;PSMB8;HLA-DRA;AIF1;LGR4;IGHM;TREX1;HLA-DPB1;IRGM;PSMB9;;LYN;CLEC5A;MMP12;CCL5;PIK3R6;RAB7B;CCL3*

Positive regulation of ERK1 and ERK2 cascade	23.077% (39/169)	9.95E-11	*MARCO;CD74;CD44;FGFR2;ATP6AP1;PTPRC;TREM2;NRP1;PDGFB;PYCARD;CCL7;CCL2;HMGCR;SLAMF1;FGF23;PTK2B;ADRA1A;PTPN22;C5AR2;CD36;GPNMB;GATA4;TLR4;PDGFC;EGFR;SCIMP;CCR1;NOD2;NPNT;GPR183;RASGRP1;CCL19;ERBB4;CSF1R;HLA-DRB1;C5AR1;TNF;CCL5;CCL3*
Positive regulation of interleukin-6 production	32% (24/75)	3.51E-10	*TYROBP;ISL1;CD74;POU2F2;TLR8;PYCARD;DDX58;IL1RL2;CD36;TLR4;TLR2;IL6R;SCIMP;SYK;NOD2;PTAFR;CLEC7A;TLR1;LILRA5;IL1RAP;MBP;AIF1;TNF;RAB7B*
Positive regulation of interleukin-1 beta production	40% (18/45)	9.21E-10	*TYROBP;ISL1;TLR8;PYCARD;GSDMD;CD36;TLR4;CASP1;NLRP12;GBP5;NLRP3;NOD2;MALT1;CLEC7A;CCL19;LILRA5;TNF;CCL3*
Leukocyte migration	29.261% (103/352)	1.84E-09	*ITGAL;SELE;CD9;CD74;CD44;PIK3CB;CD84;STK10;ADD2;SLC7A8;TREM2;HMOX1;PDGFB;RIN3;LGMN;MMP9;HCK;CORO1A;PYCARD;PIK3CG;CCL7;CCL2;PTPN6;BMP5;CCR6;ITGA4;GRB14;FN1;SLAMF1;CD48;PADI2;EPCAM;PTK2B;CCR2;CXCR4;CD244;NCKAP1L;PREX1;GPR18;ADGRE2;RAC2;MPP1;LGALS3;JCHAIN;VAV3;PTPN22;C5AR2;P2RX4;IL10;CCN3;CXCL9;ITGAX;SLC16A3;VAV1;NLRP12;S100A8;PLA2G7;TRIM55;PTPRO;EPS8;ADAM8;MCOLN2;SLC7A7;SLAMF8;FCER1G;ITGB2;IL6R;NLRP3;CD200R1;CCR1;SYK;NOD2;SLC3A2;CX3CR1;INPP5D;CXCL10;PTAFR;CXCL8;GPR183;ITGAM;PTGER4;PIK3CD;C3AR1;CCL19;OLR1;SAA1;RHOG;GBA;CSF1R;PLCB1;LCK;GCNT1;MMP1;C5AR1;SPN;AIF1;IGHM;GPSM3;TNF;PRSS56;LYN;CCL5;CCL3*
Interferon-gamma-mediated signaling pathway	37.931% (22/58)	1.89E-09	*NR1H3;CD44;IFNGR1;HCK;OAS2;GBP1;IRF5;OASL;IRF4;PARP9;IRF8;FCGR1A;IFNGR2;B2M;PTAFR;PARP14;CIITA;IRF7;HLA-DRB1;HLA-DRA;HLA-DPB1;IRGM*
Neutrophil chemotaxis	37.5% (27/72)	2.96E-09	*CD74;PIK3CG;CCL7;CCL2;NCKAP1L;PREX1;RAC2;MPP1;LGALS3;VAV3;C5AR2;CXCL9;VAV1;S100A8;FCER1G;ITGB2;SYK;NOD2;CXCL10;CXCL8;PIK3CD;C3AR1;CCL19;SAA1;C5AR1;CCL5;CCL3*
Positive regulation of interferon-gamma production	36% (18/50)	6.80E-09	*ISL1;TLR8;PYCARD;IL18R1;SLAMF1;CCR2;CD244;PTPN22;TLR4;IRF8;CYRIB;SLAMF6;CD14;CLEC7A;RASGRP1;ISG15;HLA-DPB1;TNF*

Table S12. Effects of each treatment over time on blood cell counts.

	Ctrl			Vit.D2			CHT			Lard			IR			IR/CHT		
	Pre	Post	*P-value*	Pre	Post	*P-value*	Pre	Post	*P-value*	Pre	Post	*P-value*	Pre	Post	*P-value*	Pre	Post	*P-value*
White blood cell count (K/µL)	3.44 (3.21 - 4.18)	2.89 (2.41 - 3.58)	**0.0244**	3.26 (2.95 - 3.56)	2.64 (2.04 - 3.11)	**0.0156**	4.78 (3.89 - 5.69)	4.75 (4.34 - 5.92)	0.2435	5.98 (3.46 - 6.97)	2.95 (1.74 - 3.76)	**0.0046**	5.98 (561 - 6.89)	3.89 (1.64 - 4.28)	**0.0156**	4.74 (3.89 - 5.67)	5.40 (4.87 - 6.66)	0.0833
Neutrophil count (K/µL)	1.12 (0.95 - 1.46)	0.81 (0.61 - 1.13)	**0.021**	0.79 (0.71 - 1.07)	1.03 (0.83 - 1.10)	0.6875	1.04 (0.93 - 1.26)	1.68 (1.26 - 2.28)	**0.0093**	1.20 (0.89 - 1.32)	0.87 (0.72 - 1.13)	**0.0266**	1.08 (0.97 - 1.67)	1.42 (1.06 - 1.64)	0.9375	1.12 (0.97 - 1.44)	2.21 (1.69 - 2.46)	**<0.0001**
Neutrophil percentage (%)	33.05 (26.50 - 40.70)	26.55 (23.65 - 34.55)	0.5171	28.6 (21.1 - 36.2)	47.50 (29.50 - 33.70)	0.2813	20.20 (17.90 - 30.40)	28.50 (24.50 - 39.80)	0.1202	21.20 (16.70 - 26.30)	30.00 (23.50 - 50.10)	**0.0171**	17.50 (15.60 - 29.10)	45.70 (35.10 - 66.80)	**0.0156**	23.70 (20.85 - 28.40)	40.05 (26.25 - 47.75)	**0.011**
Lymphocyte count (K/µL)	2.15 (1.91 - 2.59)	1.99 (1.65 - 2.35)	0.123	2.48 (1.82 - 2.53)	1.36 (0.97 - 2.67)	**0.0156**	3.87 (2.71 - 4.36)	3.10 (2.66 - 3.47)	0.6112	3.92 (2.32 - 5.29)	2.04 (0.55 - 2.50)	**0.0017**	4.41 (3.69 - 5.23)	1.52 (0.34 - 2.38)	**0.0313**	3.08 (2.64 - 3.82)	3.23 (2.38 - 3.63)	0.8209
Lymphocyte percentage (%)	63.15 (57.90 - 67.60)	66.20 (60.30 - 70.20)	0.3804	68.2 (60.1 - 78.1)	49.8 (43.1 - 63.30)	**0.0156**	70.00 (62.20 - 75.50)	64.00 (57.70 - 73.50)	0.6441	70.90 (65.80 - 74.40)	65.50 (38.10 - 71.50)	0.0942	70.90 (59.40 - 77.10)	35.50 (21.90 - 59.20)	**0.0469**	68.05 (59.30 - 74.80)	55.70 (46.10 - 67.75)	0.0739
Monocyte count (K/µL)	0.12 (0.08 - 0.17)	0.08 (0.03 - 0.12)	**0.0425**	0.05 (0.03 - 0.09)	0.10 (0.07 - 0.16)	**0.0469**	0.22 (0.08 - 0.35)	0.11 (0.07 - 0.19)	0.1089	0.22 (0.16 - 0.44)	0.09 (0.05 - 0.14)	**0.0005**	0.37 (0.28 - 0.43)	0.14 (0.07 - 0.29)	0.375	0.23 (0.08 - 0.35)	0.11 (0.07 - 0.18)	0.2363
Monocyte percentage (%)	3.41 (2.12 - 4.55)	2.36 (1.35 - 3.40)	0.1763	1.46 (0.97 - 3.22)	3.26 (2.93 - 3.63)	**0.0156**	5.11 (2.24 - 6.51)	1.77 (1.46 - 3.78)	0.0887	5.61 (3.52 - 7.69)	3.15 (2.18 - 3.81)	0.2163	6.38 (3.20 - 7.53)	9.01 (1.59 - 11.40)	0.5781	5.37 (1.71 - 6.75)	1.87 (1.46 - 3.61)	0.1754
Eosinophil count (K/µL)	0.00 (0.00 - 0.00)	0.00 (0.00 - 0.01)	0.1758	0.00 (0.00 - 0.02)	0.00 (0.00 - 0.00)	0.5781	0.00 (0.00 - 0.00)	0.00 (0.00 - 0.00)	0.0739	0.00 (0.00 - 0.00)	0.00 (0.00 - 0.00)	0.7695	0.00 (0.00 - 0.00)	0.00 (0.00 - 0.00)	1.000	0.00 (0.00 - 0.00)	0.00 (0.00 - 0.01)	0.3691
Eosinophil percentage (%)	0.00 (0.00 - 0.05)	0.09 (0.03 - 0.28)	0.123	0.04 (0.00 - 0.7)	0.04 (0.00 - 0.14)	0.4688	0.00 (0.00 - 0.04)	0.04 (0.02 - 0.09)	0.0554	0.01 (0.00 - 0.06)	0.02 (0.00 - 0.13)	0.625	0.00 (0.00 - 0.02)	0.00 (0.00 - 0.07)	1.000	0.00 (0.00 - 0.05)	0.02 (0.00 - 0.12)	0.4548
Basophil count (K/µL)	0.03 (0.00 - 0.09)	0.07 (0.04 - 0.13)	0.2036	0.01 (0.01 - 0.03)	0.11 (0.00 - 0.24)	0.3594	0.02 (0.00 - 0.18)	0.12 (0.05 - 0.15)	0.9358	0.09 (0.04 - 0.22)	0.05 (0.01 - 0.07)	0.0681	0.23 (0.22 - 0.33)	0.09 (0.01 - 0.11)	0.2969	0.19 (0.03 - 0.27)	0.08 (0.03 - 0.13)	0.3963
Basophil percentage (%)	0.68 (0.07 - 2.54)	2.81 (1.23 - 3.38)	0.1099	0.39 (0.19 - 0.86)	4.98 (0.14 - 0.56)	0.2188	0.59 (0.14 - 3.73)	2.15 (0.86 - 3.23)	1.000	2.49 (0.54 - 3.64)	1.43 (0.32 - 2.05)	0.6848	3.72 (3.30 - 5.49)	2.93 (0.35 - 6.69)	0.6875	3.75 (0.73 - 5.50)	1.49 (0.60 - 2.29)	0.2522
Red blood cell count (M/µL)	6.62 (6.42 - 6.88)	5.57 (5.51 - 5.86)	**0.0002**	5.70 (5.45 - 5.95)	5.53 (5.32 - 6.00)	0.9375	5.84 (5.60 - 6.20)	4.05 (3.31 - 4.59)	**<0.0001**	6.08 (5.77 - 6.27)	5.42 (5.16 - 5.68)	**0.0002**	5.85 (5.35 - 5.99)	6.03 (5.87 - 6.19)	**0.0469**	5.89 (5.67 - 6.01)	3.55 (3.10 - 4.52)	**<0.0001**
Hemoglobin (g/dL)	12.80 (12.50 - 13.30)	11.60 (11.10 - 11.80)	**0.0002**	11.40 (10.40 - 11.80)	11.80 (11.20 - 12.40)	0.1094	11.80 (10.70 - 12.00)	8.70 (7.54 - 9.37)	**<0.0001**	12.05 (11.60 - 12.30)	11.30 (10.40 - 11.50)	**0.0002**	10.90 (10.50 - 11.60)	12.10 (11.80 - 12.80)	**0.0156**	11.10 (10.80 - 11.60)	7.86 (7.39 - 9.14)	**<0.0001**
Hematocrit (%)	41.05 (40.40 - 43.50)	36.70 (35.70 - 37.70)	**0.0002**	36.90 (34.34 - 38.40)	37.70 (36.00 - 39.50)	0.1094	38.10 (34.90 - 39.50)	27.90 (24.80 - 30.50)	**<0.0001**	39.05 (37.60 - 39.80)	35.40 (34.30 - 37.00)	**0.0005**	35.10 (33.80 - 37.40)	39.10 (38.70 - 41.70)	**0.0156**	36.30 (35.50 - 38.00)	25.60 (23.55 - 29.75)	**<0.0001**
Platelet count (K/µL)	462.00 (438.00 - 517.00)	293.00 (250.00 - 307.00)	**0.0002**	362.00 (319.00 - 375.00)	281.00 (258.00 - 316.00)	**0.0156**	449.00 (387.00 - 498.00)	349.00 (305.00 - 375.00)	**0.0056**	467.00 (414.00 - 554.00)	310.00 (271.00 - 326.00)	**0.0002**	346.00 (283.00 - 451.00)	278.00 (256.00 - 361.00)	0.1563	367.50 (336.50 - 391.00)	389.50 (345.00 - 437.50)	0.2114
Mean platelet volume (fL)	4.79 (4.64 - 5.07)	3.93 (3.67 - 4.40)	**0.0002**	4.04 (3.84 - 4.29)	4.13 (3.32 - 4.46)	0.125	4.60 (4.23 - 4.82)	3.53 (3.20 - 3.80)	**0.0156**	4.55 (4.29 - 4.84)	4.05 (3.57 - 4.06)	0.0625	3.91 (3.60 - 4.32)	4.12 (3.72 - 4.60)	0.5	3.90 (3.74 - 4.20)	3.82 (3.49 - 4.15)	0.5781

Statistically significant *P-values* are in bold. Wilcoxon signed rank test was performed to determine statistical significance.

Table S13. Effects of each treatment on circulating levels of conventional lipid biomarkers, minerals and biomarkers of renal and hepatic functions.

	Ctrl			Vit.D2			CHT			Lard			IR			IR/CHT		
	Pre	Post	Delta *P-value*	Pre	Post	Delta *P-value*	Pre	Post	Delta *P-value*	Pre	Post	Delta *P-value*	Pre	Post	Delta *P-value*	Pre	Post	Delta *P-value*
Triglycerides (mg/dl)	110.08 (83.28 - 124.49)	73.84 (55.18 - 145.49)	0.688	64.65 (55.10 - 110.57)	74.64 (63.68 - 133.57)	0.469	78.98 (58.73 - 99.14)	134.65 (59.31 - 159.69)	**0.010**	61.36 §51.39 - 81.67)	69.67 (53.09 - 184.29)	0.301	65.02 (50.11 - 120.25)	129.75 (117.07 - 183.16)	**0.047**	87.15 (67.47 - 132.15)	172.98 (115.58 - 296.14)	**0.005**
Cholesterol (mg/dl)	28.04 (23.03 - 38.71)	13.99 (12.27 - 18.80)	**0.016**	35.18 (22.97 - 38.22)	14.79 (10.82 - 20.96)	**0.016**	33.07 (24.57 - 48.23)	849.50 (846.72 - 857.80)	**<0.0001**	42.00 (30.31 - 47.71)	22.44 (14.93 - 40.16)	0.052	36.68 (24.19 - 59.55)	26.88 (15.37 - 32.95)	**0.016**	37.85 (29.77 - 49.31)	841.43 (836.43 - 847.31)	**<0.0001**
LDL (mg/dl)	19.04 (14.45 - 22.02)	8.32 (6.73 - 12.65)	**0.047**	16.34 (12.87 - 23.12)	8.30 (6.98 - 10.52)	**0.016**	15.48 (12.24 - 26.99)	651.34 (550.05 - 778.28)	**<0.0001**	19.80 (13.52 - 26.90)	11.54 (7.25 - 20.58)	0.204	16.78 (9.71 - 33.47)	15.56 (7.07 - 18.40)	**0.047**	21.59 (15.47 - 28.59)	692.38 (600.64 - 804.07)	**<0.0001**
HDL (mg/dl)	15.57 (8.96 - 19.84)	6.50 (5.08 - 9.85)	**0.016**	17.01 (13.08 - 22.54)	4.93 (4.76 - 8.22)	**0.016**	21.61 (15.158 - 24.84)	110.36 (74.21 - 130.27)	**<0.0001**	25.96 (19.57 - 28.54)	13.34 (8.54 - 15.53)	**0.016**	17.84 (16.80 - 28.66)	10.78 (5.33 - 15.61)	**0.016**	20.84 (14.63 - 25.77)	129.28 (91.38 - 151.54)	**<0.0001**
Calcium (mmol/l)	3.50 (3.46 - 3.89)	3.59 (3.38 - 3.65)	0.078	3.73 (3.62 - 4.01)	3.37 (3.30 - 3.45)	**0.016**	3.74 (3.64 - 3.79)	3.62 (3.52 - 3.76)	0.061	3.76 (3.64 - 3.89)	3.61 (3.56 - 3.72)	**0.002**	3.71 (3.46 - 3.88)	3.47 (3.36 - 3.58)	**0.016**	3.87 (3.71 - 4.01)	3.66 (3.53 - 3.76)	**0.004**
Phosphorous (mg/dl)	7.33 (6.51 - 7.55)	3.02 (2.86 - 3.51)	**0.016**	7.30 (6.63 - 7.59)	2.86 (2.65 - 3.10)	**0.016**	6.29 (6.148 - 6.75)	3.65 (3.17 - 4.06)	**<0.0001**	6.69 (6.30 - 6.83)	3.68 (3.19 - 4.00)	**0.001**	6.33 (5.12 - 7.41)	2.83 (3.16 - 6.17)	**0.016**	7.89 (7.38 - 8.43)	3.37 (2.87 - 4.29)	**<0.0001**
Iron (µg/dl)	229.25 (197.42 - 269.39)	206.91 (187.38 - 246.59)	0.469	248.74 (219.88 - 277.66)	238.81 (222.11 - 275.66)	0.938	230.28 (221.65 - 251.77)	186.28 (176.92 - 276.50)	0.252	262.72 (220.19 - 276.69)	236.84 (204.65 - 263.65)	0.092	223.44 (190.68 - 259.11)	215.68 (195.51 - 224.08)	0.688	209.05 (185.36 - 251.14)	243.40 (195.16 - 278.29)	0.454
AST (U/ml)	24.11 (19.47 - 37.96)	17.8 (15.49 - 30.13)	0.219	24.21 (17.90 - 28.81)	25.30 (17.60 - 26.13)	0.688	19.17 (16.68 - 23.78)	41.56 (34.66 - 47.78)	**0.008**	19.17 (18.57 - 30.01)	18.00 (13.81 - 25.88)	0.110	31.61 (19.15 - 42.28)	31.03 (27.39 - 41.11)	0.688	27.65 (20.17 - 48.01)	37.94 (34.24 - 50.59)	0.383
ALT (U/ml)	39.87 (22.92 - 56.88)	44.31 (43.76 - 46.84)	0.813	29.62 (26.94 - 47.06)	43.97 (27.75 - 50.23)	0.078	33.19 (29.07 - 40.87)	41.39 (34.40 - 46.96)	0.129	32.64 (27.05 - 47.93)	27.97 (22.64 - 51.58)	0.424	40.26 (25.92 - 65.12)	53.81 (35.97 - 64.49)	0.297	44.20 (36.59 - 60.58)	45.12 (41.35 - 52.68)	0.375
Albumin (g/l)	40.24 (37.90 - 43.26)	40.80 (37.48 - 43.80)	0.578	41.49 (39.52 - 43.50)	40.06 (37.95 - 41.54)	0.078	42.69 (39.05 - 43.75)	40.27 (37.53 - 41.98)	**0.035**	42.02 (40.75 - 43.13)	41.58 (40.91 - 42.74)	0.519	40.56 (37.39 - 42.68)	40.67 (40.28 - 41.76)	0.688	42.08 (40.63 - 43.86)	40.24 (34.15 - 43.21)	**0.026**
Urea (mg/dl)	30.60 (27.01 - 33.87)	38.09 (35.03 - 44.11)	**0.016**	38.52 (37.83 - 43.28)	31.96 (27.93 - 34.8)	**0.016**	31.27 (29.54 - 37.70)	47.14 (40.88 - 54.47)	**0.002**	33.43 (29.39 - 36.37)	37.86 (32.99 - 40.86)	0.110	29.50 (28.05 - 32.97)	36.27 (34.03 - 37.99)	**0.031**	34.40 (32.37 - 36.99)	39.16 (35.36 - 44.31)	**0.027**
Creatinine (mg/dl)	0.76 (0.70 - 0.77)	0.87 (0.80 - 0.90)	**0.031**	0.64 (0.55 - 0.69)	0.84 (0.77 - 0.94)	**0.016**	0.67 (0.62 - 0.69)	1109.00 (0.71 - 1709.00)	**0.003**	0.68 (0.61 - 0.71)	0.76 (0.70 - 0.89)	**0.001**	0.60 (0.54 - 0.76)	0.78 (0.72 - 1.00)	**0.016**	0.69 (0.63 - 0.75)	0.64 (0.44 - 2153.00)	0.497

Statistically significant *P-values* are in bold. Wilcoxon signed rank test was performed to determine statistical significance.

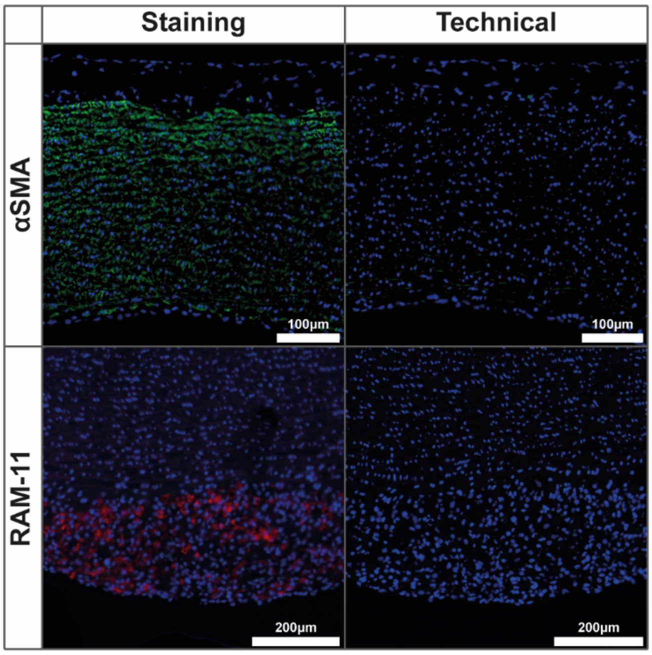

Figure S1. Negative controls for anti-αSMA and anti-RAM-11 immunofluorescent stainings. Left panels: normal staining. Right panels: technical negative controls with AF488-conjugated mouse IgG2a kappa isotype for anti-αSMA staining and with TRITC-conjugated goat anti-mouse secondary antibody without anti-RAM-11 primary antibody for anti-RAM-11 staining.

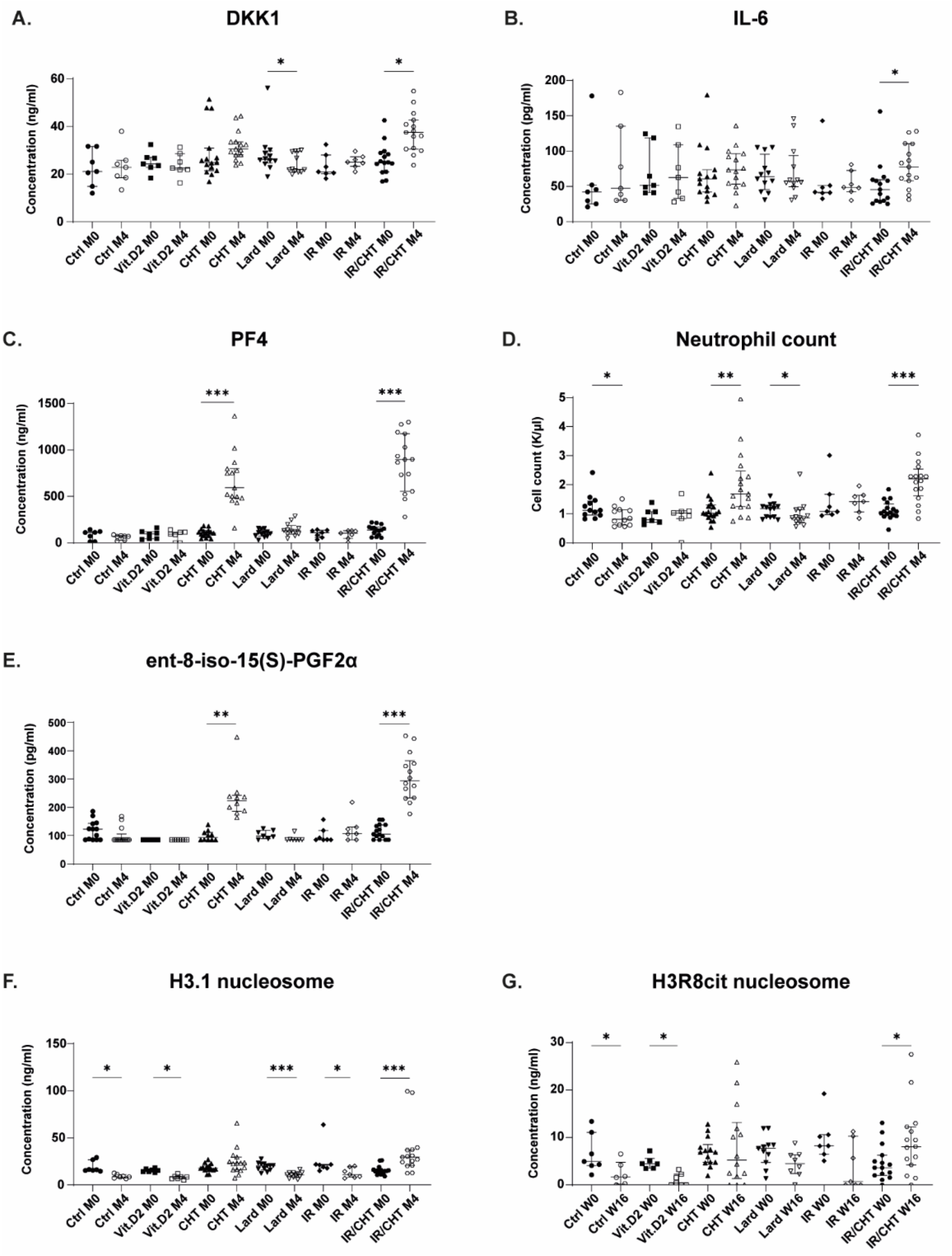

Figure S2. Effect of each treatment on levels of circulating biomarkers involved in calcification (A), platelet activation (B), inflammation (C, D), oxidative stress (E) and circulating nucleosomes (F & G). Graphs represent median and P25-P75 interquartiles. * : P-value ≤ 0.05 , ** : P-value ≤ 0.01 and *** : P-value ≤ 0.001.
